# Supplementary material for: Allosteric regulation and crystallographic fragment screening of SARS-CoV-2 NSP15 endoribonuclease
Source: Nucleic Acids Res. 2023 Apr 28;51(10):5255–70. doi: 10.1093/nar/gkad314 (PMC10250223; doi:10.1093/nar/gkad314)
Supplement: gkad314_Supplemental_Files [file gkad314_supplemental_files.zip › Supplementary datasets.zip!/134/Refine_9/01-BUSTER/Cycle-2/LIST.html]

Refining

**BUSTER 2.X**: A suite of programs for macromolecular structure refinement by maximum-likelihood and Bayesian methods  
Copyright © 1992-2020 Global Phasing Ltd.  
All rights reserved.  
Authors: G. Bricogne, E. Blanc, M. Brandl, C. Flensburg, P. Keller, W. Paciorek, P. Roversi, O. Smart, C. Vonrhein, T. Womack  

Please reference use of the software as:

- BUSTER-TNT 2.X, Global Phasing Ltd, Sheraton House, Cambridge CB3 0AX, UK

Version: 2.10.3 for linux64-ifort made on Sep 18 2020 at 01:21:52  
Licenced to: Diamond Light Source, Scientific Software  

```
---


### Parsing of the BUSTER cardfile

TITLE : autoBUSTER refinement

   Cell information

   Symmetry information

 Using CCP4 X-ray form factors.

   Missing structure chemical composition

 Using ATOM block for chemical composition.


   Shell information

### Parsing of the TNT cardfile


---


### Reading observations file

 Number of reflexions in input:  52557

      Number of accepted reflexions:  49168

      Number of rejected reflexions:      0

   Reflexions in test set:            2531

   Reflexions in working set:        46637


---


### 1. Generating initial model

 2.10 * Shannon sampling rate. NX NY NZ:   288  288  192


  Effective Shannon rates are:    2.36    2.36    2.18


   Allocated BUFFER of size:     61.172 Mb.

   Initial Geometry Information


 No missing atoms declared


   Summary of model volumes

 No missing atoms declared: no prior computed


 Computing bulk solvent model scattering


 NCSMASK PDB radius for solvent mask:     2.15 Angstrom

 running PDBSET (setting cell and spacegroup P1) ... done
 running NCSMASK [1] (creating mask, SMOOTH 1.14467) ... done
 running MAPDUMP (to check if reduction to unit cell is needed) ... done
 running MAPMASK (converting mask to map) ... done

   Generation of babslv by theta-filter

   Electron density in frg mask:  0.420 Å-1

 FOBS  put on rough absolute scale by Wilson plot :
           K        =  0.70160E+02
           B        =    -12.062

 

### Cycle 0001: MaxLik scaling and refinement


   MaxLik Scaling Cycle 0001

   Wilson plot

   Statistics in resolution bins just after scaling
```

|  |  |
| --- | --- |
|  | - Rxpct factors plot- Rxpct factors table- <Log-Lik Gain>- Ln Sigma A- Correlation Coefficients |

```
   <Log-Likelihood> of null-hypothesis

Structure Factor Amplitudes


   Average Figure of Merit

 Switching off refinement of overall temperature factor


 Added B =     0.4805 Å2 to all atoms in fragment


 Added B =     0.4805 Å2 to B_SOLV

```
gelly refinement module
 Authors (2004-2009) O. S. Smart, C. Flensburg, W. Paciorek, C. Vonrhein, 
                     M. Brandl, T. O. Womack and G. Bricogne
         (2010-2011) O. S. Smart, C. Flensburg, W. Paciorek, C. Vonrhein, 
                     T. O. Womack and G. Bricogne
         (2012-2014) O. S. Smart, C. Flensburg, W. Paciorek, C. Vonrhein, 
                     and G. Bricogne
         (2015-2020) G. Bricogne, C. Flensburg, W. Paciorek, C. Vonrhein
 Copyright © 2004-2020 Global Phasing Ltd. 
 All rights reserved.
 gelly module compilation date Sep 18 2020 01:18:35
 
 gelly uses routines from TNT geometry. References: 
     Tronrud, D. E., Ten Eyck, L. F., Matthews, B. W. (1987)
     "An Efficient General-Purpose Least-Squares Refinement Program 
     for Macromolecular Structures". Acta Crystallogr A, 43:489-501.
     Tronrud, D. E. (1997). "The TNT Refinement Package", 
     in Macromolecular Crystallography, Part B, Eds Charlie Carter 
     and Robert Sweet, Methods in Enzymology, 277:306-319.
 wildcard matching function by Julian Robichaux http://www.nsftools.com

 No -random_seed flag found. Random number stream initialised using:
2147483562
2147483398

 Control flags set to:  -autoncs -hydrogens_input_occ2zero \
                     -hydrogens_output_occ2heavy -screen \
                     100 -screen_sigma \
                     3.0 -keeppdb \
                     1000000 -taomonitor_verbose \
                     2 
 

 ############### Starting setup via TNT routines

 Setting up tnt routines including license check:

                   TNT Stereochemistry Program
 Version: 5.22.3.0
 Licence for Diamond Light Source, Scientific Software is valid
 
 Using TNT geometry routines to load the tnt control file: gelly.tnt
 Using TNT routines to load initial coordinates from file fragment.cor
 Expecting 6231 atoms

 Found     2191 "NOTE BUSTER_RESIDUE_ATOM_DATA" cards
 Have copied information for    6231 atoms from TNT to gelly's store 
 
 The TNT WEIGHT cards used by this run:
 -----WEIGHT       BOND     2.0
 -----WEIGHT       ANGLE    2.0
 -----WEIGHT       IMPROPER 0.0
 -----WEIGHT       TORSION  2.0
 -----WEIGHT       PSEUDO   0.0
 -----WEIGHT       TRIGONAL 2.0
 -----WEIGHT       PLANE    5.0
 -----WEIGHT       CONTACT  5.0
 -----WEIGHT       BCORREL  10.0
 -----WEIGHT       CHIRAL   5.0
 -----WEIGHT       NCS      50.0
 -----WEIGHT       IDEAL    4.0
 -----WEIGHT       RFACTOR  8.65
 
 Dictionary versions picked up from NOTE BUSTER_RESTRAINT_DICT cards as: 
 ------ protgeo_eh99.dat (V1.6.4.17) 20191001  STANDARD AMINO ACID DICTIONARY. BONDS AND ANGLES FROM ENGH AND HUBER EH99. OTHER VALUES BASED ON PREVIOUS TNT OR TAKEN FROM CCP4. INCLUDES HYDROGEN ATOMS.
 ------ exoticaa.dat (V1.3.4.15) 20200327  COLLECTION OF NON-STANDARD AMINO ACIDS, MAINLY EH91 WITHOUT IDEAL DISTANCE INFO
 ------ nuclgeo.dat (V1.13.4.8) 20190909
 ------ bcorrel.dat (V1.15.10.2) 20190616
 ------ contact.dat (V1.15.12.10) 20191121
 ------ idealdist_contact.dat (V1.3.4.12) 20191001  IDEAL-DISTANCE CONTACT TERM DATA AS USED IN PROLSQ. VALUES USED HERE ARE BASED ON DATA HARVESTING OF PDB AS OF 20190109.
 ------ restraints for LIG (Unknown) from cif dictionary Z1713595338.cif
 ------ restraints for CIT (CITRIC ACID) from cif dictionary CIT.cif; generated by GRADE_PDB_LIGAND 1.2.12 (October 4 2016) using MOGUL 1.7.2(RC1), CSD as538be, with quantum mechanics RM1 ((DatabaseID PDB-CIT))
 ------ assume.dat (V1.9.4.2) 20180705
 
 Have picked up spacegroup name for pdb output as 'P 63      '
 Setting up Chain/Residue arrays
 
 Have command line specification -autoncs*
     Have found       1 NCS relations
     Pairwise matching residue number and type between chains: 
                    A        B        S        W    
          A         .       99%       -        -   
          B                  .        -        -   
          S                           .        -   
          W                                    .   
       Table key--                                                       
          all   : two chains have 100% match and same number of residues 
         multi  : ditto and they contribute to a multi chain ncs cluster 
          ***%  : two chains have >80% matching residue number/type      
           -    : chains appear to be unrelated                          
      chain A     have identified ncs to     1 other chains. So will weight each autoncs by (       6.666/#)=      6.6660
      chain B     have identified ncs to     1 other chains. So will weight each autoncs by (       6.666/#)=      6.6660
      chain S     have identified ncs to     0 other chains. 
      chain W     have identified ncs to     0 other chains. 
     For .Gelly cards produced see below - after pruning
 
 Setting up BUSTER_SET groups
 Have found    91 BUSTER_SET cards
 Now silently applying automatic/dictionary set definitions. For a verbose listing use '-verbose_set' flag.
 Applying user defined set definitions:
 =====NOTE BUSTER_SET Ligand = { B|501 } --> set 'Ligand' now has    11 atoms 
 =====NOTE BUSTER_SET NotLigand = \ Ligand --> set 'NotLigand' now has  6220 atoms 
 =====NOTE BUSTER_SET ncsautoXcld = Water --> set 'ncsautoXcld' now has   691 atoms 
 =====NOTE BUSTER_SET ncsautoABset = Chain_A \ ncsautoXcld --> set 'ncsautoABset' now has  2771 atoms 
 Have defined    51 sets: 
      set:                 'All' has   6231 atoms in   1391 residues - automatically defined
      set:               'Empty' has      0 atoms in      0 residues - automatically defined
      set:            'ExoticAA' has      0 atoms in      0 residues - defined by dictionary file
      set:           'ExoticNUC' has      0 atoms in      0 residues - defined by dictionary file
      set:              'NotHET' has   5516 atoms in    698 residues - defined by dictionary file
      set:             'Chain_A' has   2771 atoms in    350 residues - automatically defined
      set:             'Chain_B' has   2769 atoms in    350 residues - automatically defined
      set:             'Chain_S' has    407 atoms in    407 residues - automatically defined
      set:             'Chain_W' has    284 atoms in    284 residues - automatically defined
      set:               'Water' has    691 atoms in    691 residues - defined by dictionary file
      set:          'StdProtein' has   5516 atoms in    698 residues - defined by dictionary file
      set:             'Protein' has   5516 atoms in    698 residues - defined by dictionary file
      set:                'Back' has   2794 atoms in    698 residues - defined by dictionary file
      set:                'Side' has   2722 atoms in    652 residues - defined by dictionary file
      set:        'RnaCanonical' has      0 atoms in      0 residues - defined by dictionary file
      set:        'DnaCanonical' has      0 atoms in      0 residues - defined by dictionary file
      set:     'RnaDnaCanonical' has      0 atoms in      0 residues - defined by dictionary file
      set:     'DnaRnaCanonical' has      0 atoms in      0 residues - defined by dictionary file
      set:         'RnaExtended' has      0 atoms in      0 residues - defined by dictionary file
      set:         'DnaExtended' has      0 atoms in      0 residues - defined by dictionary file
      set:      'RnaDnaExtended' has      0 atoms in      0 residues - defined by dictionary file
      set:      'DnaRnaExtended' has      0 atoms in      0 residues - defined by dictionary file
      set:                 'Dna' has      0 atoms in      0 residues - defined by dictionary file
      set:                 'Rna' has      0 atoms in      0 residues - defined by dictionary file
      set:              'RnaDna' has      0 atoms in      0 residues - defined by dictionary file
      set:              'DnaRna' has      0 atoms in      0 residues - defined by dictionary file
      set:             'Nucleic' has      0 atoms in      0 residues - defined by dictionary file
      set:             'Polymer' has   5516 atoms in    698 residues - defined by dictionary file
      set: 'BufferWeichenberger' has     13 atoms in      1 residues - defined by dictionary file
      set:    'BufferAdditional' has      0 atoms in      0 residues - defined by dictionary file
      set:              'Buffer' has     13 atoms in      1 residues - defined by dictionary file
      set:         'MetalAlkali' has      0 atoms in      0 residues - defined by dictionary file
      set:  'MetalAlkalineEarth' has      0 atoms in      0 residues - defined by dictionary file
      set:     'MetalTransition' has      0 atoms in      0 residues - defined by dictionary file
      set: 'MetalPostTransition' has      0 atoms in      0 residues - defined by dictionary file
      set:    'MetalLanthanides' has      0 atoms in      0 residues - defined by dictionary file
      set:               'Metal' has      0 atoms in      0 residues - defined by dictionary file
      set:         'SugarAlphaD' has      0 atoms in      0 residues - defined by dictionary file
      set:         'SugarAlphaL' has      0 atoms in      0 residues - defined by dictionary file
      set:               'Sugar' has      0 atoms in      0 residues - defined by dictionary file
      set:               'Other' has     24 atoms in      2 residues - defined by dictionary file
      set:            'Hydrogen' has      0 atoms in      0 residues - defined by dictionary file
      set:         'nonHydrogen' has   6231 atoms in   1391 residues - defined by dictionary file
      set:             'OccZero' has      0 atoms in      0 residues - defined by dictionary file
      set:          'OccNonZero' has   6231 atoms in   1391 residues - defined by dictionary file
      set:             'OccFull' has   6188 atoms in   1388 residues - defined by dictionary file
      set:          'OccNonFull' has     43 atoms in      3 residues - defined by dictionary file
      set:              'Ligand' has     11 atoms in      1 residues - user specified
      set:           'NotLigand' has   6220 atoms in   1390 residues - user specified
      set:         'ncsautoXcld' has    691 atoms in    691 residues - user specified
      set:        'ncsautoABset' has   2771 atoms in    350 residues - user specified
 No command line arguments '-occ_allhet' or '-occ_restyp' so will not setup automated occcupancy refinement
 
 Interpreting CONSTANT and FREE cards: 
 =====NOTE BUSTER_CONSTANT OCC NotLigand
 Have found and interpreted      1 CONSTANT cards in the TNT input. 
 Before any COMBINE - Number of atoms with fixed XYZ =     0
 Before any COMBINE - Number of atoms with fixed B   =     0
 Before any COMBINE - Number of atoms with fixed OCC =  6220
 Before any COMBINE - Number of atoms with fixed BIJ =   691
                            cf total number of atoms =  6231
 
 Have a total of     2 COMBINE and/or NOTE BUSTER_COMBINE cards in TNT input
 
 There are no NOTE BUSTER_COMBINE_RES cards to be found in the TNT card input 
 Interpreting COMBINE cards: 
 =====NOTE BUSTER_COMBINE OCC Ligand
 =====NOTE BUSTER_COMBINE B Ligand
 Do not have any COMBINE groups of type XYZ
 Have    1 COMBINE groups of type B
 B   combine group number     1 has    11 atoms in it:
        Residue    B|501   LIG  C01   S02   O03   O04   N05   C06   C07   C08   C09   C10   N11    
 Have    1 COMBINE groups of type OCC
 OCC combine group number     1 has    11 atoms in it:
        Residue    B|501   LIG  C01   S02   O03   O04   N05   C06   C07   C08   C09   C10   N11    
 Number of atoms with fixed/combined XYZ =     0
 Number of atoms with fixed/combined B   =    11
 Number of atoms with fixed/combined OCC =  6231
 Number of atoms with fixed/combined BIJ =   691
                cf total number of atoms =  6231
 

 ############### Working out full connectivity list


 ############### Working out equivalent atoms


 Residue with most atoms given below:
        A|58:N(TRP)         
        A|58:CA(TRP)        
        A|58:CB(TRP)        
        A|58:CG(TRP)        
        A|58:CD1(TRP)       
        A|58:NE1(TRP)       
        A|58:CE2(TRP)       
        A|58:CD2(TRP)       
        A|58:CE3(TRP)       
        A|58:CZ3(TRP)       
        A|58:CH2(TRP)       
        A|58:CZ2(TRP)       
        A|58:C(TRP)         
        A|58:O(TRP)         


 Have no '-equiv_check_plus' argument - so will take atom type into
 account when checking for equivalence of otherwise identical atoms.
 

 ############### Looking at dihedrals


 ############### Looking at chirals

 NOTE BUSTER_CHIRAL_ANGLECUT card found - read value=    -31.00 degs from it
 NOTE BUSTER_CHIRAL_SIGMA card found - read value=      1.70 degs from it
 RANGE allowed for B variables in refinement:   from      3.00000 to    300.00000

 ############### Preparing for PDB output


 ############### Processing extra restraints explicitly defined

 No 'NOTE BUSTER_TARGET' cards or -target?? args found (used to read coordinates files for soft ncs to external)
 No 'NOTE BUSTER_OCCSUM'     cards found - so not using sum of occupancy restraints
 No 'NOTE BUSTER_DISTANCE'   cards found - so not setting up any additional atom-atom distance restraints
 No 'NOTE BUSTER_UTILANGLE'  cards found - so not setting up any additional restraints on any angle
 No 'NOTE BUSTER_UTILTOR'    cards found - so not setting up any additional sinusoidal restraints on torsions
 
 --- Have some similarity definitions 
     Similarity restraints on RMSD use superposition routines from: 
     Coutsias, E.A., Seok, C., Dill, K.A.(2004)
     "Using quaternions to calculate RMSD", J. Comput. Chem., 25:1849-1857 

 Have found    1 'NOTE BUSTER_SIM_DEFINE' cards 
     Maximum number of chains involved in any SIM group (excluding the template)=     1


 ########## Interpreting card: NOTE BUSTER_SIM_DEFINE ncsautoAB ncsautoABset B
     Number of atoms in template set=  2771
     Number of related chains (other than template) in SIM set=    1
     List of chains to be restrained (excluding template)=   B   
     Pairwise RMSD, XYZ in Angstroms, for SIM group: ncsautoAB
                 A         B   
     A         -----     ----- 
     B         0.587     ----- 
     Overall RMSD,XYZ for the SIM group is     0.587 Angstroms
     Pairwise rms deviation of [individual B's minus their set/chain average] in Angs**2
                 A         B         Bavg
     A         -----     -----     97.46
     B         28.21     -----     79.35
     Overall RMSD,B   for the SIM group is    28.212 Angs**2
     The number of common atoms for the above fits:
                 A         B   
     A         -----     ----- 
     B          2758     ----- 
     (compared to the template set that has    2771 atoms in it)
     List of atoms that are absent in one or more chains for this group: 
                                    A    B   
                    C1      401     +    -
                    O1      401     +    -
                    O2      401     +    -
                    C2      401     +    -
                    C3      401     +    -
                    O7      401     +    -
                    C4      401     +    -
                    C5      401     +    -
                    O3      401     +    -
                    O4      401     +    -
                    C6      401     +    -
                    O5      401     +    -
                    O6      401     +    -
     (key  +  atom present for this chain 
           -  atom absent  for this chain)
 transforms for each of the superpositions orthogonal matrix r11 r12 r13 r21 r22 r23 r31 r32 r33 tx ty tz (PDBSET syntax): 
TRANS_AB -0.877447 -0.479375 -0.016937 -0.479452  0.877568  0.000557  0.014597  0.008609 -0.999856    33.902    32.925    15.157
 reverse transforms 
TRANS_BA -0.877447 -0.479452  0.014597 -0.479375  0.877568  0.008609 -0.016937  0.000557 -0.999856    45.312   -12.773    15.711
 
 Setting up LSSR interatomic distance restraints
 Interpreting card: NOTE BUSTER_SIM_RESTRAIN_LSSR ncsautoAB 6.666
        setting up interatomic distance pair restraints with:
        Dmin=         0.00
        Dmax=         5.50
        sigma=        0.20
        Vmax=         3.00
        weight=       6.67
        number of distance pairs defined for this set is      34775
 Have not supplied either '-sim_swap_equiv' or '-sim_swap_equiv_plus' argument 
   so will not try swaping equivalent atoms to improve NCS and/or target agreement.
 
 NCS by LSSR outlier analysis
 -autoncs option automatic NCS prune routine -------- 
 Residues pruned out by gradient condition:
          A|135     A|140     A|148     A|151     A|181     A|182     A|184
          A|185     A|186     A|189     A|216     A|218     A|219     A|220
          A|234     A|242     A|243     A|245     A|249     A|255     A|256
          A|280     A|282     A|285     A|286     A|287     A|288     A|289
          A|317     A|318     A|320     A|321     A|322     A|325     A|326
          A|327     A|328     A|329     A|330     A|340     A|341     A|342
          A|344     B|135     B|140     B|148     B|151     B|181     B|182
          B|184     B|185     B|186     B|189     B|216     B|218     B|219
          B|220     B|234     B|242     B|243     B|245     B|249     B|255
          B|256     B|280     B|282     B|285     B|286     B|287     B|288
          B|289     B|317     B|318     B|320     B|321     B|322     B|325
          B|326     B|327     B|328     B|329     B|330     B|340     B|341
          B|342     B|344
  
 Residues pruned out by function/max condition:
          A|149     A|187     A|188     A|220!    A|319     A|326!    A|346 
         A|ENDA     B|149     B|187     B|188     B|220!    B|319     B|326!
          B|346    B|ENDA 
  
 Total number of residues for which LSSR restraints have been pruned out is       98 out of      698 participating
 Number of LSSR restraints pruned out is      7116 this is   20.5% of the total           34775
  
 -autoncs .Gelly cards suitable for adaptation for manual definition/pruning
           (note auto pruning is done in a slightly different way so exact results may differ slightly)
 -autoncs option .Gelly cards also written to file  auto_gelly_cards.txt
NOTE BUSTER_SET ncsautoXcld = Water    # card added - produced by -autoncs
NOTE BUSTER_SET ncsautoXcld = ncsautoXcld + { A|135 A|140 A|148 A|149 A|151 }    # card added - produced by -autoncs
NOTE BUSTER_SET ncsautoXcld = ncsautoXcld + { A|181 A|182 A|184 A|185 A|186 }    # card added - produced by -autoncs
NOTE BUSTER_SET ncsautoXcld = ncsautoXcld + { A|187 A|188 A|189 A|216 A|218 }    # card added - produced by -autoncs
NOTE BUSTER_SET ncsautoXcld = ncsautoXcld + { A|219 A|220 A|234 A|242 A|243 }    # card added - produced by -autoncs
NOTE BUSTER_SET ncsautoXcld = ncsautoXcld + { A|245 A|249 A|255 A|256 A|280 }    # card added - produced by -autoncs
NOTE BUSTER_SET ncsautoXcld = ncsautoXcld + { A|282 A|285 A|286 A|287 A|288 }    # card added - produced by -autoncs
NOTE BUSTER_SET ncsautoXcld = ncsautoXcld + { A|289 A|317 A|318 A|319 A|320 }    # card added - produced by -autoncs
NOTE BUSTER_SET ncsautoXcld = ncsautoXcld + { A|321 A|322 A|325 A|326 A|327 }    # card added - produced by -autoncs
NOTE BUSTER_SET ncsautoXcld = ncsautoXcld + { A|328 A|329 A|330 A|340 A|341 }    # card added - produced by -autoncs
NOTE BUSTER_SET ncsautoXcld = ncsautoXcld + { A|342 A|344 A|346 A|ENDA }    # card added - produced by -autoncs
NOTE BUSTER_SET ncsautoABset = Chain_A \ ncsautoXcld    # card added - produced by -autoncs
NOTE BUSTER_SIM_DEFINE ncsautoAB ncsautoABset B    # card added - produced by -autoncs
NOTE BUSTER_SIM_RESTRAIN_LSSR ncsautoAB 6.666    # card added - produced by -autoncs

 LSSR function contribution before pruning     49512.2
                             after pruning     11388.7
 Graph of which residues pruned from LSSR: ncs_pruned.mtv
 Graph of LSSR function outliers ncs_function_outliers.mtv
 Graph of LSSR gradient outliers ncs_gradient_outliers.mtv
 LSSR - analysis of distance difference distribution for NCS
         DeltaD less than 0.5 Angs:  proportion of population= 98.45% average DeltaD of subset     0.014 Angs
 Histogram of LSSR deltaD distribution ncs_histogram.mtv
 
 Interpreting card: NOTE BUSTER_TLS_SET tls1 { A|* }
 Interpreting card: NOTE BUSTER_TLS_SET tls2 { B|* }
 Interpreting card: NOTE BUSTER_TLS_SET tls1 { A|* }
 Interpreting card: NOTE BUSTER_TLS_T   tls1     -2.75683E-01 -1.68119E-01  3.03964E-01 -3.26338E-02  4.03951E-02  3.90114E-02
 Interpreting card: NOTE BUSTER_TLS_L   tls1      1.42844E+00  1.25261E+00  4.39292E-01  2.96794E-01 -5.41106E-01  2.91932E-01
 Interpreting card: NOTE BUSTER_TLS_S   tls1      2.84530E-01 -2.02959E-01  2.55729E-01 -2.98009E-02 -1.09482E-01 -1.16911E-01 -1.71883E-02 -9.82972E-02
 Interpreting card: NOTE BUSTER_TLS_O   tls1     -2.33284E+01 -8.36882E+01  1.12128E+01
 Interpreting card: NOTE BUSTER_TLS_SET tls2 { B|* }
 Interpreting card: NOTE BUSTER_TLS_T   tls2     -3.03964E-01 -2.71348E-01  3.03964E-01  6.70261E-03  2.11698E-02 -3.20981E-02
 Interpreting card: NOTE BUSTER_TLS_L   tls2      1.79743E+00  1.32223E+00  4.53428E-01  4.28487E-01 -6.90721E-01 -7.62929E-01
 Interpreting card: NOTE BUSTER_TLS_S   tls2      6.31122E-03  1.00071E-01 -1.27050E-01  8.65256E-02  1.54687E-01  3.70038E-02 -7.61979E-02  9.77350E-02
 Interpreting card: NOTE BUSTER_TLS_O   tls2      2.20360E+01 -7.19286E+01  3.53859E+00
 Interpreting card: NOTE BUSTER_TLS_FIX tls1 ALL
 Interpreting card: NOTE BUSTER_TLS_FIX tls2 ALL


 TLS information. Total number of groups:     2


 Group   Natoms    FixRB   FixTLS   Group Tag    Group Spec
     1     2771      Y        Y     tls1    { A|* }
     2     2769      Y        Y     tls2    { B|* }
 
NOTE BUSTER_TLS_SET tls1 { A|* }
NOTE BUSTER_TLS_T   tls1     -2.75683E-01 -1.68119E-01  3.03964E-01 -3.26338E-02  4.03951E-02  3.90114E-02
NOTE BUSTER_TLS_L   tls1      1.42844E+00  1.25261E+00  4.39292E-01  2.96794E-01 -5.41106E-01  2.91932E-01
NOTE BUSTER_TLS_S   tls1      2.84530E-01 -2.02959E-01  2.55729E-01 -2.98009E-02 -1.09482E-01 -1.16911E-01 -1.71883E-02 -9.82972E-02
NOTE BUSTER_TLS_O   tls1     -2.33284E+01 -8.36882E+01  1.12128E+01
NOTE BUSTER_TLS_FIX tls1 ALL
 
NOTE BUSTER_TLS_SET tls2 { B|* }
NOTE BUSTER_TLS_T   tls2     -3.03964E-01 -2.71348E-01  3.03964E-01  6.70261E-03  2.11698E-02 -3.20981E-02
NOTE BUSTER_TLS_L   tls2      1.79743E+00  1.32223E+00  4.53428E-01  4.28487E-01 -6.90721E-01 -7.62929E-01
NOTE BUSTER_TLS_S   tls2      6.31122E-03  1.00071E-01 -1.27050E-01  8.65256E-02  1.54687E-01  3.70038E-02 -7.61979E-02  9.77350E-02
NOTE BUSTER_TLS_O   tls2      2.20360E+01 -7.19286E+01  3.53859E+00
NOTE BUSTER_TLS_FIX tls2 ALL


 TLS: mmCIF group specification begin


#
loop_
_pdbx_refine_tls_group.id
_pdbx_refine_tls_group.refine_tls_id
_pdbx_refine_tls_group.beg_PDB_ins_code
_pdbx_refine_tls_group.beg_auth_asym_id
_pdbx_refine_tls_group.beg_auth_seq_id
_pdbx_refine_tls_group.end_PDB_ins_code
_pdbx_refine_tls_group.end_auth_asym_id
_pdbx_refine_tls_group.end_auth_seq_id
_pdbx_refine_tls_group.selection
_pdbx_refine_tls_group.selection_details
   1   1 . A   -1 . A  346 . '{ A|* }'
   2   1 . A  401 . A  401 . '{ A|* }'
   3   2 . B   -1 . B  346 . '{ B|* }'
   4   2 . B  501 . B  501 . '{ B|* }'
#


 TLS: mmCIF group specification end


 Interpreting card: NOTE BUSTER_ANISO_SET Empty
 Interpreting card: NOTE BUSTER_ANISO_WEIGHT SCA  16.0
 Interpreting card: NOTE BUSTER_ANISO_WEIGHT SPH   0.6    0.6
 Interpreting card: NOTE BUSTER_ANISO_WEIGHT SIM   7.8    4.8    2.6    0
 Interpreting card: NOTE BUSTER_ANISO_WEIGHT RIG  21.5   21.5    3.7    0


 After TLS and ANISO: Number of atoms with fixed/combined xyz =     0
 After TLS and ANISO: Number of atoms with fixed/combined B   =    11
 After TLS and ANISO: Number of atoms with fixed/combined OCC =  6231
 After TLS and ANISO: Number of atoms with fixed/combined BIJ =  6231
 After TLS and ANISO: Number of atoms in a TLS group          =  5540
                                     cf total number of atoms =  6231
 

 ############### Setup state vector

 No free_vect_ulim_xyz argument found: using default of  100.000
 No free_vect_llim_xyz argument found: using default of -100.000
 Number of parameters added for B combine groups                =       1
 Number of parameters added for OCC combine groups              =       1
 Number of parameters added for TLS description                 =       0
 Number of parameters to describe molecular conformation        =   24915

 ############### Setup free vector

 Number of free parameters to be adjusted in refinement   =   24915

 ############### Setting up own geometry


 No command line argument '-tnt_fn' so will use gelly to do geometry function calculations

 ============= Call TNT functions


 ============= Get TNT symmetry information

 
      Getting symmetry operators from TNT. 
          gelly will classify symmetry using pdb-like convention: 
               SYMOP   SYMMETRY
              NNNMMM   OPERATOR
                1555   X,Y,Z
                2555   -Y,X-Y,Z
                3555   -X+Y,-X,Z
                4555   -X,-Y,1/2+Z
                5555   Y,-X+Y,1/2+Z
                6555   X-Y,X,1/2+Z
          where NNN -> operator number and MMM -> translation vector
 

 ============= Own Gelly geometry/restraints setup


 ----------- Identify bond-types


    Setting up GEOMBONDREC_TYPE_HASH* to be able to map a given bond type (e.g.
    "single") to an internal integer (GBOND_TYPE=1) etc.

    Using default  GEOMBONDREC_TYPE_HASH_STR='SING* 1 DOUB* 2 TRIP* 3 AROM* 4 DELO* 4 METAL* 5':
    to override default use a NOTE BUSTER_GEOMBONDREC_TYPE_HASH_STR gelly card.

 ----------- Classify torsion into peptide omega and other


 ----------- Setup of non-bonded terms

 Ideal-distance contact term setup:
    Restraint function constants (normally these values are from NOTE BUSTER_IDEAL_CONTACT_* cards)
    Sigma for general contacts      0.200 Angs
    Sigma for 1-4 contacts          0.100 Angs (contacts across a torsion)
    Sigma for 1-5 contacts          0.150 Angs
    Sigma for 1-N contacts          0.200 Angs
    Sigma for HBOND pairs           0.100 Angs (contacts between HB_types A and D or B's)
    Contact distance increment for each atom in HBOND          -0.150 Angs 
    Contact distance increment for H-bond acceptor              0.100 Angs 
    Contact distance increment for 1-4 with an O atom          -0.050 Angs 
    Contact distance increment for 1-4 with an N atom          -0.180 Angs 
    Contact distance increment for 1-4 with an C atom          -0.370 Angs 
    Contact distance increment for 1-4 with an S atom          -0.150 Angs 
    Contact distance increment for 1-4 with an P atom          -0.150 Angs 
    Contact distance increment for 1-4 with any other atom     -0.150 Angs 
    All atoms matched for CCP4 atom type.
    Number of CCP4 atom types used is         25 (H-bound and not H-bound counted separately)

 Setting up short/ideal-distance contacts
   Using gelly special position handling. (For TNT instead rerun with argument '-special_tnt')

   No command line argument '-special_all' specified. We will auto-generate
   EXCLUDE cards only for atom-atom contacts across symmetry (i.e. on special
   position) when this involves a contact within a residue to itself.

   Have set up non-bonded exclusions - 1-3 contacts
   Atom A|27:CA         makes the most 1-3 contacts:  6

   Have set up non-bonded exclusions - 1-4 contacts
   Atom A|27:N          makes the most 1-4 contacts:  7

   Have set up non-bonded exclusions - 1-5 contacts
   Atom A|28:C.A        makes the most 1-5 contacts: 10

 ############################################################################################################
 ###################################################### setting up contact terms
 ############################################################################################################

 WARNING . atom W|192:O(HOH)  has occupancy 1.0 but is at a special position?
 WARNING .EXCLUDE W|192 W|192
 WARNING . Generating this EXCLUDE card because an atom W|192:O(HOH) has a short contact to a 
 WARNING . symmetry copy of itself by operator:  2445 dist    0.241 Angs. occ 1.000
 WARNING . The card should ensure no bad contacts for the complete residue. If atom is at special position.... 


 ############################################################################################################
 ###################################################### setting up contact terms
 ############################################################################################################


 ============= Call Gelly versions of TNT functions

 WARNING: 1-4 contact without 1-(2,3)-4 data for A|401:O2(CIT)        and A|401:C3(CIT)        X-X
 WARNING: 1-4 contact without 1-(2,3)-4 data for A|401:C2(CIT)        and A|401:O6(CIT)        X-X
 WARNING: 1-4 contact without 1-(2,3)-4 data for A|401:O7(CIT)        and A|401:O5(CIT)        X-X
 WARNING: 1-4 contact without 1-(2,3)-4 data for A|401:C4(CIT)        and A|401:O6(CIT)        X-X
 WARNING: 1-4 contact without 1-(2,3)-4 data for B|501:S02(LIG)       and B|501:C10(LIG)       X-X

       TERM      weight      number      TNT_fn    Gelly_fn        diff
       BOND       2.000        5655    1957.004    1957.004      -0.000
       ANGL       2.000        7677    6037.630    6037.630       0.000
       IMPR       0.000           0       0.000       0.000       0.000
       TORS       2.000        1965    3490.074    3490.074      -0.000
       TRIG       2.000           0       0.000       0.000       0.000
       PLAN       5.000         962    2653.636    2653.636      -0.000
       CONT       5.000           0     276.486       0.000     276.486
       BCOR      10.000        5632   17961.228   23870.307   -5909.079


 ============= Call Gelly geometry functions

   Switching torsion functional form to sinusoidal rather than split harmonic functional.
      if you want this not to happen then rerun with '-torsharm'
      Weight for sinusoidal torsion=     2.000
   BCOR function/gradient will be split between H and non-H atoms (as for any atom pair). Use '-bcorrel_all_in_hydrogens' to change this.

 ----------- Fix B-correlation restraints

 
 Checking for bonds that do not have a bcorrel restraint defined
 We will wipe all existing BCORrels if OCC=0.0 atom(s) is/are involved. This can be' switched off via the '-bcorrel_nowipe_zeroocc' flag.
 We will wipe all existing BCORrels if only non-hydrogen atom(s) with fixed B(iso) are involved. This can be switched off via the '-bcorrel_nowipe_fixb_nonhydrogens' flag.
 We will keep all existing BCORrels if hydrogen atom(s) with fixed B(iso) is/are involved. This can be switched off via the '-bcorrel_wipe_fixb_hydrogens' flag.
 No -bcorrel_missing_sigma found. So will set sigma to     2.5 Ang**2 for any bond with no existing BCORrel
 No -bcorrel_missing_sigma_hydrogens found. So will set sigma to     1.0 Ang**2 for any bond with no existing BCORrel involving hydrogens

 No active BCORels found involving OCC=0.0 atom(s)


 No active BCORels found involving fixed-B (only) non-hydrogen atom(s)

    add a default BCOR for bond A|401:C1(CIT) A|401:C2(CIT)
    add a default BCOR for bond A|401:C1(CIT) A|401:O1(CIT)
    add a default BCOR for bond A|401:C1(CIT) A|401:O2(CIT)
    add a default BCOR for bond A|401:C2(CIT) A|401:C3(CIT)
    add a default BCOR for bond A|401:C3(CIT) A|401:C4(CIT)
    add a default BCOR for bond A|401:C3(CIT) A|401:C6(CIT)
    add a default BCOR for bond A|401:C3(CIT) A|401:O7(CIT)
    add a default BCOR for bond A|401:C4(CIT) A|401:C5(CIT)
    add a default BCOR for bond A|401:C5(CIT) A|401:O3(CIT)
    add a default BCOR for bond A|401:C5(CIT) A|401:O4(CIT)
    add a default BCOR for bond A|401:C6(CIT) A|401:O5(CIT)
    add a default BCOR for bond A|401:C6(CIT) A|401:O6(CIT)

 Have added a total of        12 BCORrels for bonds which do not already have a BCORrel


 Have skipped a total of        11 bonds to setup BCORrels where both atoms have constant/fixed B(iso)


 ----------- Deactivate hydrogen-free planes if hydrogen atoms present

 A total of       0 planes have been deactivated because they are duplicated hydrogen-free and with-hydrogen forms.

 ----------- Handle peptide omega angle(s)

      Torsion angles split into peptide omega and other
         Number of peptide omega angles is       698
         Number of other torsion is             1267
      Peptide omega angles are monitored rather than directly restrained (PLANE used instead).

 ----------- Fix cis-PRO

 Now apply the fix to get correct restraints for cis PRO residues
   Have got      11 bonds records and      26 angles
   from GEOMETRY cards for the special PROC residue
   Weight for ideal-distance contact term=     4.000

 ----------- Prepare SSBOND/LINK for output PDB file


 ----------- Prepare for QM


 ----------- Prepare symmetry in utility distance restraints


 ############### Setting up Rhofit contacts (if needed)

 No ligand-(fixed protein) contact term will be used

 ############### Working out initial function and gradient


   MaxLik Scaling Cycle 0002
 
 Initial functional value = 4.185956E+05 rms gradient=      255.976
 X-ray contribution to function value = 3.575948E+05
 Geometry contribution (inc ncs)      =      61000.8
 Geometry function summary
     term                           number     weight       rms        GooF      function     fn/numb
     ================================================================================================
     BOND bond lengths (angs)       5655       2.000      0.0084       0.416      1957.00       0.346
     ANGL bond angles (degs)        7677       2.000        1.05       0.627      6037.63       0.786
     TORS split-harmonic tors       1267       0.000       18.64       1.174         0.00       0.000
     SINTOR sinusoidal tors         1267       2.000       18.64                  2617.70       2.066
     PLAN general plane (angs)       962       5.000      0.0149       0.743      2653.64       2.758
     BCORrelations (angs^2)         5644      10.000       1.823       0.651     23887.61       4.232
     CONT (bad contacts)               0       5.000                                 0.00
     IDEAL(ideal-dist contact)      5136       4.000                             12411.03
     CHIRAL (gelly semiharmon)       743       5.000                                47.43
     SIM similarity restraint for NCS and/or TARGET                              11388.74
     OCCSUM restraint on sum of occupanies                                           0.00       as none defined
     DISTAN utility restraints interatomic distance                                  0.00       as none defined
     UTILANGLE utility angle restraints                                              0.00       as none defined
     UTILTOR utility restraints on torsion angles                                    0.00       as none defined
     Peptide omega torsion angles                           2.98 degs - monitor only
 
 WARNING: Have |delta/sigma| deviations > 5.0 sigma. Number of outliers for each term: 
 WARNING:         1 idealD contacts. Worst     5.1 sigs     2.60 Angs B|49:CD2(LEU)=W|269:O(HOH) symm: 1555=4544
 WARNING:         2 Bcorrel.      Worst is    22.9 sigs   -56.93 Angs^2 B|-1:CA=CB (ALA)

 Weighted rms      0.0084         1.052  (temporary rms bond and angle for BUSTER to pick up!)
 Weighted intra-residual rms(bond) for StdProtein (excluding H)      0.0081
 Weighted intra-residual rms(bond) for Buffer     (excluding H)      0.0026

 ############### Running initial check


 Initial Geometry Sanity Check

 Check for duplicate bonds: no duplicates found
 
 Check for duplicate or missing bond angles: all are OK
 Check for bond between an atom and itself not involving symmetry:  all are OK
   There are no bad contacts where Delta/sigma is greater than     6.5
   Worst contact dist=   2.598 std=   3.603 sigma=   0.198 abs(Delta/sigma)=    5.077 ats B|49:CD2 (LEU) W|269:O (HOH)
   It is likely that the geometry libraries, sequence info and links match the input molecule
 GEOMETRY CHECK OVERALL:  PASS


 ############### Creating geometry/screen report


 ############### Option "tao" - run Tao optimizer

 TAO: Toolkit for Advanced Optimization. Reference: 
     Munson, T., Sarich, J., Wild, S., Benson, S., McInnes, L.C. (2018)
     "TAO User Manual (Revision 3.9)", Mathematics and Computer Science
     Division, Argonne National Laboratory
     ANL/MCS-TM-322, http://www.mcs.anl.gov/petsc/
 
 Have found -keeppdb flag so will write out a .pdb file every 1000000 its during opt
 Will apply TAO optimization 
 Maximum number of iterations      100
 Convergence test on Grms       4.0000 (-glim)
 Limit on rms displacement to original position (rmsD2init): NONE test inactive (-dlim)

    analysing argument no 0 : -autoncs with j=0
    analysing argument no 1 : -hydrogens_input_occ2zero with j=0
    analysing argument no 2 : -hydrogens_output_occ2heavy with j=0
    analysing argument no 3 : -screen with j=0
    analysing argument no 4 : 100 with j=0
    analysing argument no 5 : -screen_sigma with j=0
    analysing argument no 6 : 3.0 with j=0
    analysing argument no 7 : -keeppdb with j=0
    analysing argument no 8 : 1000000 with j=0
    analysing argument no 9 : -taomonitor_verbose with j=0
    found argument starting with "-tao" : -taomonitor_verbose
    analysing argument no 10 : 2 with j=1
    found value after -tao/-options_/-viewer_ argument : "2"
 PETSC/TAO: setting option "-taomonitor_verbose" = "2"


   MaxLik Scaling Cycle 0003
   Iters     Tot_Funct    Xray_Funct    Geom_Funct          Grms     rmsD2init     maxD2init             R         Rfree           LLG       LLGfree     rms(bond)    rms(angle)       Opttag_H
   Iters             K   B_IMPF_FRAG        K_MISS   B_TEMP_MISS   K_IMPF_MISS   B_IMPF_MISS        K_SOLV   B_TEMP_SOLV   K_IMPF_SOLV   B_IMPF_SOLV        B_TEMP     B_TEMP_11     B_TEMP_12     B_TEMP_22     B_TEMP_13     B_TEMP_23     B_TEMP_33      K_WILSON      B_WILSON      FGCYCLES       Scatag_H
   Iters          BOND         ANGLE      IMPROPER       TORSION    SINTORSION      TRIGONAL        PLANAR  BCORRELATION       CONTACT     IDEALDIST        CHIRAL    SIMILARITY       OCCSSUM  UTILDISTANCE     UTILANGLE   UTILTORSION        RHOFIT        ADP-01        ADP-02        ADP-03        ADP-04        ADP-05        ADP-06        ADP-07        ADP-08        ADP-09        ADP-10      RHOFITPC            QM       Funtag_H
       0  4.185956E+05  3.575948E+05       61000.8       255.976  0.000000E+00  0.000000E+00      0.197385      0.226739  0.000000E+00  0.000000E+00    0.00835040       1.05218       Opttag_D
       0       85.3864       1.71959       1.00000  0.000000E+00       1.00000  0.000000E+00      0.930334       77.0512       1.00000       27.8874  0.000000E+00      0.887703  0.000000E+00      0.887703  0.000000E+00  0.000000E+00      -1.77541       85.7100       1.33362        2       Scatag_D
       0       1957.00       6037.63  0.000000E+00  0.000000E+00       2617.70  0.000000E+00       2653.64       23887.6  0.000000E+00       12411.0       47.4271       11388.7 -1.000000E+10 -1.000000E+10 -1.000000E+10 -1.000000E+10 -1.000000E+10  0.000000E+00  0.000000E+00  0.000000E+00  0.000000E+00  0.000000E+00  0.000000E+00  0.000000E+00  0.000000E+00  0.000000E+00  0.000000E+00  0.000000E+00  0.000000E+00       Funtag_D
   Geometry Restraint Deviations SCREEN Information

 

   MaxLik Scaling Cycle 0004

   MaxLik Scaling Cycle 0005

   MaxLik Scaling Cycle 0006

   Wilson plot

   Statistics in resolution bins after refinement cycle 0001
```


|  |  |
| --- | --- |
|  | - Rxpct factors plot- Rxpct factors table- <Log-Lik Gain>- Ln Sigma A- Correlation Coefficients |


```
   Iters     Tot_Funct    Xray_Funct    Geom_Funct          Grms     rmsD2init     maxD2init             R         Rfree           LLG       LLGfree     rms(bond)    rms(angle)
   Iters             K   B_IMPF_FRAG        K_MISS   B_TEMP_MISS   K_IMPF_MISS   B_IMPF_MISS        K_SOLV   B_TEMP_SOLV   K_IMPF_SOLV   B_IMPF_SOLV        B_TEMP     B_TEMP_11     B_TEMP_12     B_TEMP_22     B_TEMP_13     B_TEMP_23     B_TEMP_33      K_WILSON      B_WILSON      FGCYCLES
   Iters          BOND         ANGLE      IMPROPER       TORSION    SINTORSION      TRIGONAL        PLANAR  BCORRELATION       CONTACT     IDEALDIST        CHIRAL    SIMILARITY       OCCSSUM  UTILDISTANCE     UTILANGLE   UTILTORSION        RHOFIT        ADP-01        ADP-02        ADP-03        ADP-04        ADP-05        ADP-06        ADP-07        ADP-08        ADP-09        ADP-10      RHOFITPC            QM
       1  4.139251E+05  3.570050E+05       56920.1       276.401    0.00131279     0.0249679      0.197197      0.226470    0.00146125    0.00224955    0.00817650       1.03917       Opttag_D
       1       85.3120       1.68836       1.00000  0.000000E+00       1.00000  0.000000E+00      0.930269       76.6813       1.00000       27.9535  0.000000E+00      0.899298  0.000000E+00      0.899298  0.000000E+00  0.000000E+00      -1.79860       85.7100       1.33362        3       Scatag_D
       1       1876.34       5889.20  0.000000E+00  0.000000E+00       2617.53  0.000000E+00       2635.24       20159.9  0.000000E+00       12363.9       46.4844       11331.5 -1.000000E+10 -1.000000E+10 -1.000000E+10 -1.000000E+10 -1.000000E+10  0.000000E+00  0.000000E+00  0.000000E+00  0.000000E+00  0.000000E+00  0.000000E+00  0.000000E+00  0.000000E+00  0.000000E+00  0.000000E+00  0.000000E+00  0.000000E+00       Funtag_D
   Geometry Restraint Deviations SCREEN Information

 

   MaxLik Scaling Cycle 0007

   Wilson plot

   Statistics in resolution bins after refinement cycle 0002
```


|  |  |
| --- | --- |
|  | - Rxpct factors plot- Rxpct factors table- <Log-Lik Gain>- Ln Sigma A- Correlation Coefficients |


```
   Iters     Tot_Funct    Xray_Funct    Geom_Funct          Grms     rmsD2init     maxD2init             R         Rfree           LLG       LLGfree     rms(bond)    rms(angle)
   Iters             K   B_IMPF_FRAG        K_MISS   B_TEMP_MISS   K_IMPF_MISS   B_IMPF_MISS        K_SOLV   B_TEMP_SOLV   K_IMPF_SOLV   B_IMPF_SOLV        B_TEMP     B_TEMP_11     B_TEMP_12     B_TEMP_22     B_TEMP_13     B_TEMP_23     B_TEMP_33      K_WILSON      B_WILSON      FGCYCLES
   Iters          BOND         ANGLE      IMPROPER       TORSION    SINTORSION      TRIGONAL        PLANAR  BCORRELATION       CONTACT     IDEALDIST        CHIRAL    SIMILARITY       OCCSSUM  UTILDISTANCE     UTILANGLE   UTILTORSION        RHOFIT        ADP-01        ADP-02        ADP-03        ADP-04        ADP-05        ADP-06        ADP-07        ADP-08        ADP-09        ADP-10      RHOFITPC            QM
       2  4.080075E+05  3.567389E+05       51268.7       183.319    0.00384264     0.0547256      0.197107      0.226763    0.00212110    0.00143957    0.00807682       1.04548       Opttag_D
       2       85.1932       1.66808       1.00000  0.000000E+00       1.00000  0.000000E+00      0.930140       76.2751       1.00000       27.8587  0.000000E+00      0.899946  0.000000E+00      0.899946  0.000000E+00  0.000000E+00      -1.79989       85.7100       1.33362        1       Scatag_D
       2       1830.87       5961.01  0.000000E+00  0.000000E+00       2617.63  0.000000E+00       2598.84       14700.5  0.000000E+00       12266.0       46.9220       11246.9 -1.000000E+10 -1.000000E+10 -1.000000E+10 -1.000000E+10 -1.000000E+10  0.000000E+00  0.000000E+00  0.000000E+00  0.000000E+00  0.000000E+00  0.000000E+00  0.000000E+00  0.000000E+00  0.000000E+00  0.000000E+00  0.000000E+00  0.000000E+00       Funtag_D
   Geometry Restraint Deviations SCREEN Information

 
       3  4.056792E+05  3.566680E+05       49011.2       130.314    0.00402445     0.0454192      0.197062      0.226780    0.00229674    0.00127395    0.00803739       1.05935       Opttag_D
       3       85.1680       1.66366       1.00000  0.000000E+00       1.00000  0.000000E+00      0.930059       76.2314       1.00000       27.8290  0.000000E+00      0.903864  0.000000E+00      0.903864  0.000000E+00  0.000000E+00      -1.80773       85.7100       1.33362        1       Scatag_D
       3       1813.04       6120.20  0.000000E+00  0.000000E+00       2617.09  0.000000E+00       2594.73       12333.9  0.000000E+00       12249.5       46.5079       11236.3 -1.000000E+10 -1.000000E+10 -1.000000E+10 -1.000000E+10 -1.000000E+10  0.000000E+00  0.000000E+00  0.000000E+00  0.000000E+00  0.000000E+00  0.000000E+00  0.000000E+00  0.000000E+00  0.000000E+00  0.000000E+00  0.000000E+00  0.000000E+00       Funtag_D
       4  4.044903E+05  3.566234E+05       47867.0       56.3197    0.00404191     0.0443344      0.197040      0.226751    0.00240731    0.00141517    0.00795141       1.03470       Opttag_D
       4       85.1601       1.66122       1.00000  0.000000E+00       1.00000  0.000000E+00      0.930031       76.2170       1.00000       27.8263  0.000000E+00      0.905991  0.000000E+00      0.905991  0.000000E+00  0.000000E+00      -1.81198       85.7100       1.33362        1       Scatag_D
       4       1774.46       5838.69  0.000000E+00  0.000000E+00       2617.47  0.000000E+00       2593.52       11522.9  0.000000E+00       12242.9       46.3433       11230.7 -1.000000E+10 -1.000000E+10 -1.000000E+10 -1.000000E+10 -1.000000E+10  0.000000E+00  0.000000E+00  0.000000E+00  0.000000E+00  0.000000E+00  0.000000E+00  0.000000E+00  0.000000E+00  0.000000E+00  0.000000E+00  0.000000E+00  0.000000E+00       Funtag_D
       5  4.039918E+05  3.565469E+05       47444.8       40.1774    0.00450463     0.0458335      0.197009      0.226759    0.00259685    0.00141526    0.00792229       1.03211       Opttag_D
       5       85.1333       1.65603       1.00000  0.000000E+00       1.00000  0.000000E+00      0.929972       76.1463       1.00000       27.8048  0.000000E+00      0.908488  0.000000E+00      0.908488  0.000000E+00  0.000000E+00      -1.81698       85.7100       1.33362        1       Scatag_D
       5       1761.48       5809.49  0.000000E+00  0.000000E+00       2617.55  0.000000E+00       2587.42       11182.6  0.000000E+00       12221.9       46.3209       11218.1 -1.000000E+10 -1.000000E+10 -1.000000E+10 -1.000000E+10 -1.000000E+10  0.000000E+00  0.000000E+00  0.000000E+00  0.000000E+00  0.000000E+00  0.000000E+00  0.000000E+00  0.000000E+00  0.000000E+00  0.000000E+00  0.000000E+00  0.000000E+00       Funtag_D
       6  4.030580E+05  3.563054E+05       46752.6       46.7410    0.00628444     0.0635295      0.196916      0.226817    0.00319547    0.00123469    0.00786760       1.03063       Opttag_D
       6       85.0364       1.63888       1.00000  0.000000E+00       1.00000  0.000000E+00      0.929751       75.8986       1.00000       27.7186  0.000000E+00      0.917534  0.000000E+00      0.917534  0.000000E+00  0.000000E+00      -1.83507       85.7100       1.33362        1       Scatag_D
       6       1737.25       5792.88  0.000000E+00  0.000000E+00       2617.72  0.000000E+00       2567.18       10663.0  0.000000E+00       12146.8       46.2766       11181.5 -1.000000E+10 -1.000000E+10 -1.000000E+10 -1.000000E+10 -1.000000E+10  0.000000E+00  0.000000E+00  0.000000E+00  0.000000E+00  0.000000E+00  0.000000E+00  0.000000E+00  0.000000E+00  0.000000E+00  0.000000E+00  0.000000E+00  0.000000E+00       Funtag_D
       7  4.027345E+05  3.559393E+05       46795.2       128.492    0.00907692     0.0997934      0.196773      0.226858    0.00410300    0.00102171    0.00799105       1.07934       Opttag_D
       7       84.8920       1.61389       1.00000  0.000000E+00       1.00000  0.000000E+00      0.929423       75.5478       1.00000       27.5836  0.000000E+00      0.931548  0.000000E+00      0.931548  0.000000E+00  0.000000E+00      -1.86310       85.7100       1.33362        1       Scatag_D
       7       1792.19       6353.39  0.000000E+00  0.000000E+00       2618.78  0.000000E+00       2543.31       10254.4  0.000000E+00       12039.6       45.6163       11147.9 -1.000000E+10 -1.000000E+10 -1.000000E+10 -1.000000E+10 -1.000000E+10  0.000000E+00  0.000000E+00  0.000000E+00  0.000000E+00  0.000000E+00  0.000000E+00  0.000000E+00  0.000000E+00  0.000000E+00  0.000000E+00  0.000000E+00  0.000000E+00       Funtag_D
       8  4.018580E+05  3.558579E+05       46000.1       57.4500    0.00973907      0.109188      0.196742      0.226878    0.00430477  9.387501E-04    0.00783563       1.03225       Opttag_D
       8       84.8568       1.60815       1.00000  0.000000E+00       1.00000  0.000000E+00      0.929341       75.4644       1.00000       27.5499  0.000000E+00      0.934979  0.000000E+00      0.934979  0.000000E+00  0.000000E+00      -1.86996       85.7100       1.33362        1       Scatag_D
       8       1723.16       5811.02  0.000000E+00  0.000000E+00       2618.49  0.000000E+00       2538.44       10115.7  0.000000E+00       12012.1       45.4877       11135.8 -1.000000E+10 -1.000000E+10 -1.000000E+10 -1.000000E+10 -1.000000E+10  0.000000E+00  0.000000E+00  0.000000E+00  0.000000E+00  0.000000E+00  0.000000E+00  0.000000E+00  0.000000E+00  0.000000E+00  0.000000E+00  0.000000E+00  0.000000E+00       Funtag_D
       9  4.012786E+05  3.556820E+05       45596.6       45.0835     0.0108017      0.124461      0.196665      0.226873    0.00474090  9.537014E-04    0.00780999       1.02749       Opttag_D
       9       84.8002       1.59751       1.00000  0.000000E+00       1.00000  0.000000E+00      0.929216       75.3321       1.00000       27.4981  0.000000E+00      0.940808  0.000000E+00      0.940808  0.000000E+00  0.000000E+00      -1.88162       85.7100       1.33362        1       Scatag_D
       9       1711.90       5757.54  0.000000E+00  0.000000E+00       2618.45  0.000000E+00       2532.13       9838.48  0.000000E+00       11969.8       45.2109       11123.1 -1.000000E+10 -1.000000E+10 -1.000000E+10 -1.000000E+10 -1.000000E+10  0.000000E+00  0.000000E+00  0.000000E+00  0.000000E+00  0.000000E+00  0.000000E+00  0.000000E+00  0.000000E+00  0.000000E+00  0.000000E+00  0.000000E+00  0.000000E+00       Funtag_D
      10  4.004979E+05  3.553526E+05       45145.4       65.6157     0.0133180      0.159411      0.196529      0.226903    0.00555755  9.128024E-04    0.00782327       1.03212       Opttag_D
      10       84.6796       1.57630       1.00000  0.000000E+00       1.00000  0.000000E+00      0.928949       75.0422       1.00000       27.3879  0.000000E+00      0.953488  0.000000E+00      0.953488  0.000000E+00  0.000000E+00      -1.90698       85.7100       1.33362        1       Scatag_D
      10       1717.73       5809.61  0.000000E+00  0.000000E+00       2618.95  0.000000E+00       2520.62       9449.98  0.000000E+00       11880.8       44.7943       11102.9 -1.000000E+10 -1.000000E+10 -1.000000E+10 -1.000000E+10 -1.000000E+10  0.000000E+00  0.000000E+00  0.000000E+00  0.000000E+00  0.000000E+00  0.000000E+00  0.000000E+00  0.000000E+00  0.000000E+00  0.000000E+00  0.000000E+00  0.000000E+00       Funtag_D
      11  3.996056E+05  3.549610E+05       44644.6       100.012     0.0175766      0.218164      0.196379      0.227032    0.00652815  7.416821E-04    0.00792246       1.05479       Opttag_D
      11       84.5023       1.54811       1.00000  0.000000E+00       1.00000  0.000000E+00      0.928562       74.5969       1.00000       27.2191  0.000000E+00      0.972629  0.000000E+00      0.972629  0.000000E+00  0.000000E+00      -1.94526       85.7100       1.33362        1       Scatag_D
      11       1761.56       6067.60  0.000000E+00  0.000000E+00       2619.77  0.000000E+00       2509.91       8806.42  0.000000E+00       11748.5       44.2277       11086.5 -1.000000E+10 -1.000000E+10 -1.000000E+10 -1.000000E+10 -1.000000E+10  0.000000E+00  0.000000E+00  0.000000E+00  0.000000E+00  0.000000E+00  0.000000E+00  0.000000E+00  0.000000E+00  0.000000E+00  0.000000E+00  0.000000E+00  0.000000E+00       Funtag_D
      12  3.988317E+05  3.548148E+05       44016.9       71.6684     0.0222700      0.281862      0.196387      0.227254    0.00689049  1.827920E-04    0.00793257       1.03718       Opttag_D
      12       84.3422       1.52906       1.00000  0.000000E+00       1.00000  0.000000E+00      0.928198       74.1563       1.00000       27.0644  0.000000E+00      0.990824  0.000000E+00      0.990824  0.000000E+00  0.000000E+00      -1.98165       85.7100       1.33362        1       Scatag_D
      12       1766.06       5866.71  0.000000E+00  0.000000E+00       2621.82  0.000000E+00       2506.57       8500.92  0.000000E+00       11625.9       43.7343       11085.2 -1.000000E+10 -1.000000E+10 -1.000000E+10 -1.000000E+10 -1.000000E+10  0.000000E+00  0.000000E+00  0.000000E+00  0.000000E+00  0.000000E+00  0.000000E+00  0.000000E+00  0.000000E+00  0.000000E+00  0.000000E+00  0.000000E+00  0.000000E+00       Funtag_D
      13  3.983511E+05  3.547571E+05       43594.1       38.0936     0.0225305      0.286746      0.196356      0.227253    0.00703363  2.672911E-04    0.00786147       1.02440       Opttag_D
      13       84.3309       1.52626       1.00000  0.000000E+00       1.00000  0.000000E+00      0.928204       74.1176       1.00000       27.0422  0.000000E+00      0.990247  0.000000E+00      0.990247  0.000000E+00  0.000000E+00      -1.98049       85.7100       1.33362        1       Scatag_D
      13       1734.54       5722.99  0.000000E+00  0.000000E+00       2621.78  0.000000E+00       2506.00       8277.58  0.000000E+00       11615.4       43.6256       11072.1 -1.000000E+10 -1.000000E+10 -1.000000E+10 -1.000000E+10 -1.000000E+10  0.000000E+00  0.000000E+00  0.000000E+00  0.000000E+00  0.000000E+00  0.000000E+00  0.000000E+00  0.000000E+00  0.000000E+00  0.000000E+00  0.000000E+00  0.000000E+00       Funtag_D
      14  3.979629E+05  3.546513E+05       43311.6       42.6803     0.0231482      0.297169      0.196296      0.227260    0.00729579  3.730114E-04    0.00781775       1.02474       Opttag_D
      14       84.3074       1.52094       1.00000  0.000000E+00       1.00000  0.000000E+00      0.928203       74.0397       1.00000       26.9973  0.000000E+00      0.989239  0.000000E+00      0.989239  0.000000E+00  0.000000E+00      -1.97848       85.7100       1.33362        1       Scatag_D
      14       1715.30       5726.75  0.000000E+00  0.000000E+00       2621.55  0.000000E+00       2505.55       8048.86  0.000000E+00       11593.9       43.4605       11056.2 -1.000000E+10 -1.000000E+10 -1.000000E+10 -1.000000E+10 -1.000000E+10  0.000000E+00  0.000000E+00  0.000000E+00  0.000000E+00  0.000000E+00  0.000000E+00  0.000000E+00  0.000000E+00  0.000000E+00  0.000000E+00  0.000000E+00  0.000000E+00       Funtag_D
      15  3.975959E+05  3.545141E+05       43081.8       50.1347     0.0247436      0.320812      0.196232      0.227311    0.00763602  3.406972E-04    0.00781828       1.02451       Opttag_D
      15       84.2561       1.51226       1.00000  0.000000E+00       1.00000  0.000000E+00      0.928146       73.8797       1.00000       26.9174  0.000000E+00      0.990237  0.000000E+00      0.990237  0.000000E+00  0.000000E+00      -1.98047       85.7100       1.33362        1       Scatag_D
      15       1715.53       5724.23  0.000000E+00  0.000000E+00       2621.42  0.000000E+00       2506.05       7877.15  0.000000E+00       11551.1       43.2052       11043.1 -1.000000E+10 -1.000000E+10 -1.000000E+10 -1.000000E+10 -1.000000E+10  0.000000E+00  0.000000E+00  0.000000E+00  0.000000E+00  0.000000E+00  0.000000E+00  0.000000E+00  0.000000E+00  0.000000E+00  0.000000E+00  0.000000E+00  0.000000E+00       Funtag_D
      16  3.970594E+05  3.542535E+05       42805.9       61.2524     0.0288266      0.379085      0.196113      0.227469    0.00828180  2.073893E-05    0.00787567       1.02711       Opttag_D
      16       84.1423       1.49446       1.00000  0.000000E+00       1.00000  0.000000E+00      0.927999       73.5139       1.00000       26.7358  0.000000E+00      0.992801  0.000000E+00      0.992801  0.000000E+00  0.000000E+00      -1.98560       85.7100       1.33362        1       Scatag_D
      16       1740.81       5753.27  0.000000E+00  0.000000E+00       2621.16  0.000000E+00       2511.18       7652.60  0.000000E+00       11456.8       42.8033       11027.3 -1.000000E+10 -1.000000E+10 -1.000000E+10 -1.000000E+10 -1.000000E+10  0.000000E+00  0.000000E+00  0.000000E+00  0.000000E+00  0.000000E+00  0.000000E+00  0.000000E+00  0.000000E+00  0.000000E+00  0.000000E+00  0.000000E+00  0.000000E+00       Funtag_D
      17  3.965983E+05  3.541315E+05       42466.8       38.9181     0.0311703      0.411827      0.196063      0.227583    0.00858434 -3.393468E-04    0.00787891       1.01958       Opttag_D
      17       84.0856       1.48588       1.00000  0.000000E+00       1.00000  0.000000E+00      0.927902       73.3341       1.00000       26.6401  0.000000E+00      0.994578  0.000000E+00      0.994578  0.000000E+00  0.000000E+00      -1.98916       85.7100       1.33362        1       Scatag_D
      17       1742.25       5669.31  0.000000E+00  0.000000E+00       2620.97  0.000000E+00       2513.72       7448.41  0.000000E+00       11407.8       42.6539       11021.7 -1.000000E+10 -1.000000E+10 -1.000000E+10 -1.000000E+10 -1.000000E+10  0.000000E+00  0.000000E+00  0.000000E+00  0.000000E+00  0.000000E+00  0.000000E+00  0.000000E+00  0.000000E+00  0.000000E+00  0.000000E+00  0.000000E+00  0.000000E+00       Funtag_D
      18  3.962448E+05  3.540246E+05       42220.3       35.8775     0.0328402      0.434959      0.196015      0.227670    0.00884935 -6.730989E-04    0.00787238       1.01926       Opttag_D
      18       84.0482       1.47945       1.00000  0.000000E+00       1.00000  0.000000E+00      0.927819       73.2227       1.00000       26.5735  0.000000E+00      0.995765  0.000000E+00      0.995765  0.000000E+00  0.000000E+00      -1.99153       85.7100       1.33362        1       Scatag_D
      18       1739.36       5665.73  0.000000E+00  0.000000E+00       2620.76  0.000000E+00       2513.55       7249.43  0.000000E+00       11374.4       42.5898       11014.4 -1.000000E+10 -1.000000E+10 -1.000000E+10 -1.000000E+10 -1.000000E+10  0.000000E+00  0.000000E+00  0.000000E+00  0.000000E+00  0.000000E+00  0.000000E+00  0.000000E+00  0.000000E+00  0.000000E+00  0.000000E+00  0.000000E+00  0.000000E+00       Funtag_D
      19  3.958925E+05  3.538588E+05       42033.7       41.5410     0.0355200      0.471464      0.195943      0.227806    0.00926043   -0.00124752    0.00788079       1.01967       Opttag_D
      19       83.9964       1.46993       1.00000  0.000000E+00       1.00000  0.000000E+00      0.927693       73.0633       1.00000       26.4711  0.000000E+00      0.996401  0.000000E+00      0.996401  0.000000E+00  0.000000E+00      -1.99280       85.7100       1.33362        1       Scatag_D
      19       1743.08       5670.31  0.000000E+00  0.000000E+00       2620.24  0.000000E+00       2512.72       7115.20  0.000000E+00       11325.8       42.6983       11003.6 -1.000000E+10 -1.000000E+10 -1.000000E+10 -1.000000E+10 -1.000000E+10  0.000000E+00  0.000000E+00  0.000000E+00  0.000000E+00  0.000000E+00  0.000000E+00  0.000000E+00  0.000000E+00  0.000000E+00  0.000000E+00  0.000000E+00  0.000000E+00       Funtag_D
      20  3.956676E+05  3.536911E+05       41976.5       54.7764     0.0389587      0.517036      0.195869      0.228003    0.00967596   -0.00206685    0.00792330       1.02409       Opttag_D
      20       83.9425       1.46008       1.00000  0.000000E+00       1.00000  0.000000E+00      0.927545       72.8837       1.00000       26.3502  0.000000E+00      0.995489  0.000000E+00      0.995489  0.000000E+00  0.000000E+00      -1.99098       85.7100       1.33362        1       Scatag_D
      20       1761.93       5719.52  0.000000E+00  0.000000E+00       2619.51  0.000000E+00       2511.95       7058.51  0.000000E+00       11269.1       43.0070       10993.0 -1.000000E+10 -1.000000E+10 -1.000000E+10 -1.000000E+10 -1.000000E+10  0.000000E+00  0.000000E+00  0.000000E+00  0.000000E+00  0.000000E+00  0.000000E+00  0.000000E+00  0.000000E+00  0.000000E+00  0.000000E+00  0.000000E+00  0.000000E+00       Funtag_D
      21  3.954029E+05  3.536375E+05       41765.4       29.8061     0.0394843      0.523633      0.195839      0.228004    0.00980889   -0.00214363    0.00789524       1.01754       Opttag_D
      21       83.9372       1.45797       1.00000  0.000000E+00       1.00000  0.000000E+00      0.927526       72.8636       1.00000       26.3309  0.000000E+00      0.993619  0.000000E+00      0.993619  0.000000E+00  0.000000E+00      -1.98724       85.7100       1.33362        1       Scatag_D
      21       1749.48       5646.56  0.000000E+00  0.000000E+00       2619.21  0.000000E+00       2509.82       6948.66  0.000000E+00       11260.2       43.0691       10988.3 -1.000000E+10 -1.000000E+10 -1.000000E+10 -1.000000E+10 -1.000000E+10  0.000000E+00  0.000000E+00  0.000000E+00  0.000000E+00  0.000000E+00  0.000000E+00  0.000000E+00  0.000000E+00  0.000000E+00  0.000000E+00  0.000000E+00  0.000000E+00       Funtag_D
      22  3.951268E+05  3.535233E+05       41603.5       29.1834     0.0411042      0.543854      0.195776      0.228049     0.0100921   -0.00245298    0.00789302       1.01735       Opttag_D
      22       83.9211       1.45301       1.00000  0.000000E+00       1.00000  0.000000E+00      0.927468       72.7968       1.00000       26.2760  0.000000E+00      0.989288  0.000000E+00      0.989288  0.000000E+00  0.000000E+00      -1.97858       85.7100       1.33362        1       Scatag_D
      22       1748.49       5644.54  0.000000E+00  0.000000E+00       2618.36  0.000000E+00       2506.10       6826.68  0.000000E+00       11235.3       43.1960       10980.8 -1.000000E+10 -1.000000E+10 -1.000000E+10 -1.000000E+10 -1.000000E+10  0.000000E+00  0.000000E+00  0.000000E+00  0.000000E+00  0.000000E+00  0.000000E+00  0.000000E+00  0.000000E+00  0.000000E+00  0.000000E+00  0.000000E+00  0.000000E+00       Funtag_D
      23  3.948900E+05  3.533856E+05       41504.4       35.0179     0.0437851      0.576876      0.195705      0.228179     0.0104334   -0.00304795    0.00791193       1.01842       Opttag_D
      23       83.8974       1.44649       1.00000  0.000000E+00       1.00000  0.000000E+00      0.927372       72.6876       1.00000       26.1943  0.000000E+00      0.983840  0.000000E+00      0.983840  0.000000E+00  0.000000E+00      -1.96768       85.7100       1.33362        1       Scatag_D
      23       1756.88       5656.35  0.000000E+00  0.000000E+00       2617.30  0.000000E+00       2503.07       6755.17  0.000000E+00       11198.5       43.2036       10974.0 -1.000000E+10 -1.000000E+10 -1.000000E+10 -1.000000E+10 -1.000000E+10  0.000000E+00  0.000000E+00  0.000000E+00  0.000000E+00  0.000000E+00  0.000000E+00  0.000000E+00  0.000000E+00  0.000000E+00  0.000000E+00  0.000000E+00  0.000000E+00       Funtag_D
      24  3.947427E+05  3.531904E+05       41552.2       58.5885     0.0483870      0.631357      0.195602      0.228389     0.0109172   -0.00412357    0.00792938       1.02599       Opttag_D
      24       83.8740       1.43772       1.00000  0.000000E+00       1.00000  0.000000E+00      0.927217       72.5284       1.00000       26.0721  0.000000E+00      0.973333  0.000000E+00      0.973333  0.000000E+00  0.000000E+00      -1.94667       85.7100       1.33362        1       Scatag_D
      24       1764.64       5740.78  0.000000E+00  0.000000E+00       2615.17  0.000000E+00       2499.01       6779.92  0.000000E+00       11145.2       42.9732       10964.5 -1.000000E+10 -1.000000E+10 -1.000000E+10 -1.000000E+10 -1.000000E+10  0.000000E+00  0.000000E+00  0.000000E+00  0.000000E+00  0.000000E+00  0.000000E+00  0.000000E+00  0.000000E+00  0.000000E+00  0.000000E+00  0.000000E+00  0.000000E+00       Funtag_D
      25  3.945011E+05  3.531557E+05       41345.4       26.2420     0.0488896      0.636947      0.195577      0.228403     0.0110032   -0.00423431    0.00789655       1.01680       Opttag_D
      25       83.8745       1.43655       1.00000  0.000000E+00       1.00000  0.000000E+00      0.927195       72.5182       1.00000       26.0593  0.000000E+00      0.972506  0.000000E+00      0.972506  0.000000E+00  0.000000E+00      -1.94501       85.7100       1.33362        1       Scatag_D
      25       1750.06       5638.45  0.000000E+00  0.000000E+00       2615.02  0.000000E+00       2497.65       6701.01  0.000000E+00       11139.0       42.7737       10961.5 -1.000000E+10 -1.000000E+10 -1.000000E+10 -1.000000E+10 -1.000000E+10  0.000000E+00  0.000000E+00  0.000000E+00  0.000000E+00  0.000000E+00  0.000000E+00  0.000000E+00  0.000000E+00  0.000000E+00  0.000000E+00  0.000000E+00  0.000000E+00       Funtag_D
      26  3.943698E+05  3.531011E+05       41268.7       23.1796     0.0493380      0.641405      0.195537      0.228397     0.0111386   -0.00430367    0.00788031       1.01673       Opttag_D
      26       83.8788       1.43511       1.00000  0.000000E+00       1.00000  0.000000E+00      0.927173       72.5189       1.00000       26.0473  0.000000E+00      0.971182  0.000000E+00      0.971182  0.000000E+00  0.000000E+00      -1.94236       85.7100       1.33362        1       Scatag_D
      26       1742.86       5637.68  0.000000E+00  0.000000E+00       2614.66  0.000000E+00       2495.25       6644.47  0.000000E+00       11133.3       42.5223       10957.9 -1.000000E+10 -1.000000E+10 -1.000000E+10 -1.000000E+10 -1.000000E+10  0.000000E+00  0.000000E+00  0.000000E+00  0.000000E+00  0.000000E+00  0.000000E+00  0.000000E+00  0.000000E+00  0.000000E+00  0.000000E+00  0.000000E+00  0.000000E+00       Funtag_D
      27  3.941946E+05  3.529803E+05       41214.3       27.1737     0.0511101      0.660099      0.195447      0.228460     0.0114380   -0.00467166    0.00787190       1.01731       Opttag_D
      27       83.8883       1.43176       1.00000  0.000000E+00       1.00000  0.000000E+00      0.927108       72.5002       1.00000       26.0054  0.000000E+00      0.966460  0.000000E+00      0.966460  0.000000E+00  0.000000E+00      -1.93292       85.7100       1.33362        1       Scatag_D
      27       1739.15       5644.11  0.000000E+00  0.000000E+00       2613.62  0.000000E+00       2491.01       6616.31  0.000000E+00       11115.7       42.1961       10952.2 -1.000000E+10 -1.000000E+10 -1.000000E+10 -1.000000E+10 -1.000000E+10  0.000000E+00  0.000000E+00  0.000000E+00  0.000000E+00  0.000000E+00  0.000000E+00  0.000000E+00  0.000000E+00  0.000000E+00  0.000000E+00  0.000000E+00  0.000000E+00       Funtag_D
      28  3.941379E+05  3.527854E+05       41352.5       64.8449     0.0544985      0.694353      0.195289      0.228597     0.0119212   -0.00538576    0.00790273       1.02865       Opttag_D
      28       83.9203       1.42760       1.00000  0.000000E+00       1.00000  0.000000E+00      0.927005       72.4922       1.00000       25.9335  0.000000E+00      0.956643  0.000000E+00      0.956643  0.000000E+00  0.000000E+00      -1.91329       85.7100       1.33362        1       Scatag_D
      28       1752.80       5770.60  0.000000E+00  0.000000E+00       2611.61  0.000000E+00       2484.97       6656.43  0.000000E+00       11086.7       42.0087       10947.4 -1.000000E+10 -1.000000E+10 -1.000000E+10 -1.000000E+10 -1.000000E+10  0.000000E+00  0.000000E+00  0.000000E+00  0.000000E+00  0.000000E+00  0.000000E+00  0.000000E+00  0.000000E+00  0.000000E+00  0.000000E+00  0.000000E+00  0.000000E+00       Funtag_D
      29  3.939070E+05  3.527586E+05       41148.3       26.3781     0.0551847      0.701420      0.195267      0.228631     0.0119875   -0.00553488    0.00786544       1.01616       Opttag_D
      29       83.9263       1.42709       1.00000  0.000000E+00       1.00000  0.000000E+00      0.926992       72.4866       1.00000       25.9210  0.000000E+00      0.954611  0.000000E+00      0.954611  0.000000E+00  0.000000E+00      -1.90922       85.7100       1.33362        1       Scatag_D
      29       1736.29       5631.33  0.000000E+00  0.000000E+00       2611.42  0.000000E+00       2484.36       6616.62  0.000000E+00       11081.6       41.9826       10944.7 -1.000000E+10 -1.000000E+10 -1.000000E+10 -1.000000E+10 -1.000000E+10  0.000000E+00  0.000000E+00  0.000000E+00  0.000000E+00  0.000000E+00  0.000000E+00  0.000000E+00  0.000000E+00  0.000000E+00  0.000000E+00  0.000000E+00  0.000000E+00       Funtag_D
      30  3.937989E+05  3.527140E+05       41084.9       18.0525     0.0560651      0.710060      0.195226      0.228669     0.0120981   -0.00570079    0.00785842       1.01529       Opttag_D
      30       83.9381       1.42650       1.00000  0.000000E+00       1.00000  0.000000E+00      0.926982       72.4886       1.00000       25.9047  0.000000E+00      0.951564  0.000000E+00      0.951564  0.000000E+00  0.000000E+00      -1.90313       85.7100       1.33362        1       Scatag_D
      30       1733.20       5621.67  0.000000E+00  0.000000E+00       2611.00  0.000000E+00       2483.30       6576.34  0.000000E+00       11075.0       42.0052       10942.4 -1.000000E+10 -1.000000E+10 -1.000000E+10 -1.000000E+10 -1.000000E+10  0.000000E+00  0.000000E+00  0.000000E+00  0.000000E+00  0.000000E+00  0.000000E+00  0.000000E+00  0.000000E+00  0.000000E+00  0.000000E+00  0.000000E+00  0.000000E+00       Funtag_D
      31  3.936529E+05  3.526002E+05       41052.7       23.6512     0.0584017      0.732313      0.195118      0.228764     0.0123803   -0.00614364    0.00787054       1.01628       Opttag_D
      31       83.9727       1.42533       1.00000  0.000000E+00       1.00000  0.000000E+00      0.926953       72.5023       1.00000       25.8642  0.000000E+00      0.943656  0.000000E+00      0.943656  0.000000E+00  0.000000E+00      -1.88731       85.7100       1.33362        1       Scatag_D
      31       1738.55       5632.69  0.000000E+00  0.000000E+00       2610.03  0.000000E+00       2481.07       6549.76  0.000000E+00       11059.1       41.9677       10939.5 -1.000000E+10 -1.000000E+10 -1.000000E+10 -1.000000E+10 -1.000000E+10  0.000000E+00  0.000000E+00  0.000000E+00  0.000000E+00  0.000000E+00  0.000000E+00  0.000000E+00  0.000000E+00  0.000000E+00  0.000000E+00  0.000000E+00  0.000000E+00       Funtag_D
      32  3.935315E+05  3.524280E+05       41103.5       42.3613     0.0619165      0.771587      0.194953      0.228899     0.0128070   -0.00674102    0.00789670       1.02136       Opttag_D
      32       84.0356       1.42443       1.00000  0.000000E+00       1.00000  0.000000E+00      0.926932       72.5426       1.00000       25.8105  0.000000E+00      0.931862  0.000000E+00      0.931862  0.000000E+00  0.000000E+00      -1.86372       85.7100       1.33362        1       Scatag_D
      32       1750.12       5689.09  0.000000E+00  0.000000E+00       2608.78  0.000000E+00       2479.41       6558.15  0.000000E+00       11038.4       41.6517       10937.9 -1.000000E+10 -1.000000E+10 -1.000000E+10 -1.000000E+10 -1.000000E+10  0.000000E+00  0.000000E+00  0.000000E+00  0.000000E+00  0.000000E+00  0.000000E+00  0.000000E+00  0.000000E+00  0.000000E+00  0.000000E+00  0.000000E+00  0.000000E+00       Funtag_D
      33  3.933821E+05  3.523477E+05       41034.4       20.5636     0.0639021      0.797268      0.194872      0.228972     0.0130062   -0.00711664    0.00788945       1.01585       Opttag_D
      33       84.0736       1.42457       1.00000  0.000000E+00       1.00000  0.000000E+00      0.926922       72.5694       1.00000       25.7841  0.000000E+00      0.925675  0.000000E+00      0.925675  0.000000E+00  0.000000E+00      -1.85135       85.7100       1.33362        1       Scatag_D
      33       1746.91       5627.84  0.000000E+00  0.000000E+00       2608.32  0.000000E+00       2479.29       6563.48  0.000000E+00       11029.9       41.4766       10937.2 -1.000000E+10 -1.000000E+10 -1.000000E+10 -1.000000E+10 -1.000000E+10  0.000000E+00  0.000000E+00  0.000000E+00  0.000000E+00  0.000000E+00  0.000000E+00  0.000000E+00  0.000000E+00  0.000000E+00  0.000000E+00  0.000000E+00  0.000000E+00       Funtag_D
      34  3.932763E+05  3.522657E+05       41010.6       14.7741     0.0653914      0.816097      0.194796      0.229002     0.0132094   -0.00733821    0.00788968       1.01489       Opttag_D
      34       84.1079       1.42458       1.00000  0.000000E+00       1.00000  0.000000E+00      0.926927       72.6009       1.00000       25.7645  0.000000E+00      0.920949  0.000000E+00      0.920949  0.000000E+00  0.000000E+00      -1.84190       85.7100       1.33362        1       Scatag_D
      34       1747.01       5617.23  0.000000E+00  0.000000E+00       2608.18  0.000000E+00       2479.25       6558.18  0.000000E+00       11023.3       41.2605       10936.2 -1.000000E+10 -1.000000E+10 -1.000000E+10 -1.000000E+10 -1.000000E+10  0.000000E+00  0.000000E+00  0.000000E+00  0.000000E+00  0.000000E+00  0.000000E+00  0.000000E+00  0.000000E+00  0.000000E+00  0.000000E+00  0.000000E+00  0.000000E+00       Funtag_D
      35  3.931729E+05  3.521578E+05       41015.1       21.2925     0.0672139      0.838492      0.194693      0.229026     0.0134770   -0.00759218    0.00788914       1.01574       Opttag_D
      35       84.1540       1.42480       1.00000  0.000000E+00       1.00000  0.000000E+00      0.926946       72.6463       1.00000       25.7406  0.000000E+00      0.915131  0.000000E+00      0.915131  0.000000E+00  0.000000E+00      -1.83026       85.7100       1.33362        1       Scatag_D
      35       1746.77       5626.61  0.000000E+00  0.000000E+00       2608.10  0.000000E+00       2479.01       6563.69  0.000000E+00       11015.8       41.0582       10934.1 -1.000000E+10 -1.000000E+10 -1.000000E+10 -1.000000E+10 -1.000000E+10  0.000000E+00  0.000000E+00  0.000000E+00  0.000000E+00  0.000000E+00  0.000000E+00  0.000000E+00  0.000000E+00  0.000000E+00  0.000000E+00  0.000000E+00  0.000000E+00       Funtag_D
      36  3.930791E+05  3.519687E+05       41110.4       39.4843     0.0705681      0.877862      0.194506      0.229074     0.0139457   -0.00807246    0.00791757       1.01900       Opttag_D
      36       84.2462       1.42624       1.00000  0.000000E+00       1.00000  0.000000E+00      0.927001       72.7424       1.00000       25.6986  0.000000E+00      0.904310  0.000000E+00      0.904310  0.000000E+00  0.000000E+00      -1.80862       85.7100       1.33362        1       Scatag_D
      36       1759.38       5662.80  0.000000E+00  0.000000E+00       2608.74  0.000000E+00       2478.72       6622.01  0.000000E+00       11005.9       40.8818       10932.0 -1.000000E+10 -1.000000E+10 -1.000000E+10 -1.000000E+10 -1.000000E+10  0.000000E+00  0.000000E+00  0.000000E+00  0.000000E+00  0.000000E+00  0.000000E+00  0.000000E+00  0.000000E+00  0.000000E+00  0.000000E+00  0.000000E+00  0.000000E+00       Funtag_D
      37  3.929382E+05  3.519164E+05       41021.8       18.4996     0.0711502      0.883882      0.194454      0.229068     0.0140752   -0.00811963    0.00788470       1.01424       Opttag_D
      37       84.2669       1.42649       1.00000  0.000000E+00       1.00000  0.000000E+00      0.927026       72.7673       1.00000       25.6884  0.000000E+00      0.901931  0.000000E+00      0.901931  0.000000E+00  0.000000E+00      -1.80386       85.7100       1.33362        1       Scatag_D
      37       1744.81       5610.05  0.000000E+00  0.000000E+00       2608.91  0.000000E+00       2478.02       6607.41  0.000000E+00       11003.2       40.9288       10928.4 -1.000000E+10 -1.000000E+10 -1.000000E+10 -1.000000E+10 -1.000000E+10  0.000000E+00  0.000000E+00  0.000000E+00  0.000000E+00  0.000000E+00  0.000000E+00  0.000000E+00  0.000000E+00  0.000000E+00  0.000000E+00  0.000000E+00  0.000000E+00       Funtag_D
      38  3.928230E+05  3.518429E+05       40980.1       16.6224     0.0717068      0.888604      0.194383      0.229043     0.0142576   -0.00812223    0.00787021       1.01407       Opttag_D
      38       84.2921       1.42671       1.00000  0.000000E+00       1.00000  0.000000E+00      0.927066       72.8013       1.00000       25.6743  0.000000E+00      0.898889  0.000000E+00      0.898889  0.000000E+00  0.000000E+00      -1.79778       85.7100       1.33362        1       Scatag_D
      38       1738.40       5608.14  0.000000E+00  0.000000E+00       2609.38  0.000000E+00       2476.27       6581.94  0.000000E+00       10999.9       41.0783       10924.9 -1.000000E+10 -1.000000E+10 -1.000000E+10 -1.000000E+10 -1.000000E+10  0.000000E+00  0.000000E+00  0.000000E+00  0.000000E+00  0.000000E+00  0.000000E+00  0.000000E+00  0.000000E+00  0.000000E+00  0.000000E+00  0.000000E+00  0.000000E+00       Funtag_D
      39  3.927176E+05  3.517421E+05       40975.5       21.1029     0.0728334      0.898954      0.194280      0.229015     0.0145074   -0.00819718    0.00786819       1.01442       Opttag_D
      39       84.3353       1.42758       1.00000  0.000000E+00       1.00000  0.000000E+00      0.927118       72.8580       1.00000       25.6536  0.000000E+00      0.893783  0.000000E+00      0.893783  0.000000E+00  0.000000E+00      -1.78757       85.7100       1.33362        1       Scatag_D
      39       1737.51       5612.02  0.000000E+00  0.000000E+00       2610.30  0.000000E+00       2474.27       6581.68  0.000000E+00       10996.0       41.1432       10922.6 -1.000000E+10 -1.000000E+10 -1.000000E+10 -1.000000E+10 -1.000000E+10  0.000000E+00  0.000000E+00  0.000000E+00  0.000000E+00  0.000000E+00  0.000000E+00  0.000000E+00  0.000000E+00  0.000000E+00  0.000000E+00  0.000000E+00  0.000000E+00       Funtag_D
      40  3.926940E+05  3.515426E+05       41151.4       51.8996     0.0754147      0.921040      0.194068      0.228947     0.0150018   -0.00833937    0.00791498       1.02357       Opttag_D
      40       84.4334       1.43024       1.00000  0.000000E+00       1.00000  0.000000E+00      0.927207       72.9956       1.00000       25.6136  0.000000E+00      0.883387  0.000000E+00      0.883387  0.000000E+00  0.000000E+00      -1.76677       85.7100       1.33362        1       Scatag_D
      40       1758.23       5713.69  0.000000E+00  0.000000E+00       2612.05  0.000000E+00       2470.24       6645.62  0.000000E+00       10988.8       41.0564       10921.7 -1.000000E+10 -1.000000E+10 -1.000000E+10 -1.000000E+10 -1.000000E+10  0.000000E+00  0.000000E+00  0.000000E+00  0.000000E+00  0.000000E+00  0.000000E+00  0.000000E+00  0.000000E+00  0.000000E+00  0.000000E+00  0.000000E+00  0.000000E+00       Funtag_D
      41  3.925241E+05  3.515250E+05       40999.1       16.0848     0.0757663      0.924295      0.194047      0.228950     0.0150455   -0.00838856    0.00786998       1.01272       Opttag_D
      41       84.4447       1.43061       1.00000  0.000000E+00       1.00000  0.000000E+00      0.927212       73.0101       1.00000       25.6099  0.000000E+00      0.882238  0.000000E+00      0.882238  0.000000E+00  0.000000E+00      -1.76448       85.7100       1.33362        1       Scatag_D
      41       1738.30       5593.21  0.000000E+00  0.000000E+00       2612.66  0.000000E+00       2469.78       6634.59  0.000000E+00       10989.3       40.9905       10920.3 -1.000000E+10 -1.000000E+10 -1.000000E+10 -1.000000E+10 -1.000000E+10  0.000000E+00  0.000000E+00  0.000000E+00  0.000000E+00  0.000000E+00  0.000000E+00  0.000000E+00  0.000000E+00  0.000000E+00  0.000000E+00  0.000000E+00  0.000000E+00       Funtag_D
      42  3.924731E+05  3.514991E+05       40974.0       11.1447     0.0760960      0.926872      0.194024      0.228936     0.0151097   -0.00839811    0.00786898       1.01188       Opttag_D
      42       84.4555       1.43072       1.00000  0.000000E+00       1.00000  0.000000E+00      0.927210       73.0270       1.00000       25.6061  0.000000E+00      0.881332  0.000000E+00      0.881332  0.000000E+00  0.000000E+00      -1.76266       85.7100       1.33362        1       Scatag_D
      42       1737.86       5584.00  0.000000E+00  0.000000E+00       2613.05  0.000000E+00       2468.49       6621.46  0.000000E+00       10989.2       40.9053       10919.1 -1.000000E+10 -1.000000E+10 -1.000000E+10 -1.000000E+10 -1.000000E+10  0.000000E+00  0.000000E+00  0.000000E+00  0.000000E+00  0.000000E+00  0.000000E+00  0.000000E+00  0.000000E+00  0.000000E+00  0.000000E+00  0.000000E+00  0.000000E+00       Funtag_D
      43  3.923962E+05  3.514201E+05       40976.1       16.3600     0.0771798      0.934894      0.193947      0.228890     0.0153056   -0.00844181    0.00787302       1.01187       Opttag_D
      43       84.4945       1.43162       1.00000  0.000000E+00       1.00000  0.000000E+00      0.927222       73.0857       1.00000       25.5936  0.000000E+00      0.878030  0.000000E+00      0.878030  0.000000E+00  0.000000E+00      -1.75606       85.7100       1.33362        1       Scatag_D
      43       1739.64       5583.88  0.000000E+00  0.000000E+00       2614.09  0.000000E+00       2465.69       6625.90  0.000000E+00       10989.2       40.7638       10916.9 -1.000000E+10 -1.000000E+10 -1.000000E+10 -1.000000E+10 -1.000000E+10  0.000000E+00  0.000000E+00  0.000000E+00  0.000000E+00  0.000000E+00  0.000000E+00  0.000000E+00  0.000000E+00  0.000000E+00  0.000000E+00  0.000000E+00  0.000000E+00       Funtag_D
      44  3.923010E+05  3.513187E+05       40982.2       19.1112     0.0787481      0.945297      0.193842      0.228823     0.0155568   -0.00853016    0.00787384       1.01123       Opttag_D
      44       84.5528       1.43344       1.00000  0.000000E+00       1.00000  0.000000E+00      0.927246       73.1694       1.00000       25.5779  0.000000E+00      0.873329  0.000000E+00      0.873329  0.000000E+00  0.000000E+00      -1.74666       85.7100       1.33362        1       Scatag_D
      44       1740.01       5576.76  0.000000E+00  0.000000E+00       2615.49  0.000000E+00       2462.72       6642.51  0.000000E+00       10991.3       40.5652       10912.9 -1.000000E+10 -1.000000E+10 -1.000000E+10 -1.000000E+10 -1.000000E+10  0.000000E+00  0.000000E+00  0.000000E+00  0.000000E+00  0.000000E+00  0.000000E+00  0.000000E+00  0.000000E+00  0.000000E+00  0.000000E+00  0.000000E+00  0.000000E+00       Funtag_D
      45  3.922705E+05  3.511547E+05       41115.9       37.1878     0.0822210      0.966442      0.193651      0.228695     0.0159636   -0.00883816    0.00788391       1.01231       Opttag_D
      45       84.6838       1.43926       1.00000  0.000000E+00       1.00000  0.000000E+00      0.927322       73.3478       1.00000       25.5525  0.000000E+00      0.863201  0.000000E+00      0.863201  0.000000E+00  0.000000E+00      -1.72640       85.7100       1.33362        1       Scatag_D
      45       1744.46       5588.68  0.000000E+00  0.000000E+00       2618.57  0.000000E+00       2459.43       6756.10  0.000000E+00       10999.8       40.1933       10908.6 -1.000000E+10 -1.000000E+10 -1.000000E+10 -1.000000E+10 -1.000000E+10  0.000000E+00  0.000000E+00  0.000000E+00  0.000000E+00  0.000000E+00  0.000000E+00  0.000000E+00  0.000000E+00  0.000000E+00  0.000000E+00  0.000000E+00  0.000000E+00       Funtag_D
      46  3.921510E+05  3.511629E+05       40988.1       13.2696     0.0819190      0.963626      0.193665      0.228694     0.0159431   -0.00880527    0.00785691       1.00892       Opttag_D
      46       84.6740       1.43869       1.00000  0.000000E+00       1.00000  0.000000E+00      0.927320       73.3297       1.00000       25.5540  0.000000E+00      0.863866  0.000000E+00      0.863866  0.000000E+00  0.000000E+00      -1.72773       85.7100       1.33362        1       Scatag_D
      46       1732.53       5551.39  0.000000E+00  0.000000E+00       2618.19  0.000000E+00       2459.55       6681.80  0.000000E+00       10998.6       40.1631       10905.9 -1.000000E+10 -1.000000E+10 -1.000000E+10 -1.000000E+10 -1.000000E+10  0.000000E+00  0.000000E+00  0.000000E+00  0.000000E+00  0.000000E+00  0.000000E+00  0.000000E+00  0.000000E+00  0.000000E+00  0.000000E+00  0.000000E+00  0.000000E+00       Funtag_D
      47  3.921058E+05  3.511566E+05       40949.2       11.2139     0.0819862      0.962778      0.193661      0.228685     0.0159588   -0.00882215    0.00784680       1.00872       Opttag_D
      47       84.6786       1.43881       1.00000  0.000000E+00       1.00000  0.000000E+00      0.927331       73.3283       1.00000       25.5535  0.000000E+00      0.863402  0.000000E+00      0.863402  0.000000E+00  0.000000E+00      -1.72680       85.7100       1.33362        1       Scatag_D
      47       1728.07       5549.14  0.000000E+00  0.000000E+00       2618.13  0.000000E+00       2459.44       6653.00  0.000000E+00       10998.2       40.0990       10903.2 -1.000000E+10 -1.000000E+10 -1.000000E+10 -1.000000E+10 -1.000000E+10  0.000000E+00  0.000000E+00  0.000000E+00  0.000000E+00  0.000000E+00  0.000000E+00  0.000000E+00  0.000000E+00  0.000000E+00  0.000000E+00  0.000000E+00  0.000000E+00       Funtag_D
      48  3.920402E+05  3.511129E+05       40927.3       13.6743     0.0829400      0.965683      0.193617      0.228643     0.0160670   -0.00893790    0.00783791       1.00842       Opttag_D
      48       84.7185       1.44056       1.00000  0.000000E+00       1.00000  0.000000E+00      0.927368       73.3658       1.00000       25.5483  0.000000E+00      0.860268  0.000000E+00      0.860268  0.000000E+00  0.000000E+00      -1.72054       85.7100       1.33362        1       Scatag_D
      48       1724.16       5545.83  0.000000E+00  0.000000E+00       2618.88  0.000000E+00       2458.85       6641.44  0.000000E+00       11000.1       39.9049       10898.2 -1.000000E+10 -1.000000E+10 -1.000000E+10 -1.000000E+10 -1.000000E+10  0.000000E+00  0.000000E+00  0.000000E+00  0.000000E+00  0.000000E+00  0.000000E+00  0.000000E+00  0.000000E+00  0.000000E+00  0.000000E+00  0.000000E+00  0.000000E+00       Funtag_D
      49  3.919960E+05  3.510222E+05       40973.7       30.8568     0.0849415      0.976181      0.193529      0.228563     0.0162918   -0.00915320    0.00784093       1.01000       Opttag_D
      49       84.7991       1.44389       1.00000  0.000000E+00       1.00000  0.000000E+00      0.927429       73.4471       1.00000       25.5410  0.000000E+00      0.854820  0.000000E+00      0.854820  0.000000E+00  0.000000E+00      -1.70964       85.7100       1.33362        1       Scatag_D
      49       1725.49       5563.24  0.000000E+00  0.000000E+00       2620.41  0.000000E+00       2458.06       6670.95  0.000000E+00       11004.7       39.6230       10891.2 -1.000000E+10 -1.000000E+10 -1.000000E+10 -1.000000E+10 -1.000000E+10  0.000000E+00  0.000000E+00  0.000000E+00  0.000000E+00  0.000000E+00  0.000000E+00  0.000000E+00  0.000000E+00  0.000000E+00  0.000000E+00  0.000000E+00  0.000000E+00       Funtag_D
      50  3.919179E+05  3.509892E+05       40928.7       15.5082     0.0857599      0.980878      0.193495      0.228532     0.0163737   -0.00923471    0.00782936       1.00780       Opttag_D
      50       84.8313       1.44528       1.00000  0.000000E+00       1.00000  0.000000E+00      0.927449       73.4813       1.00000       25.5388  0.000000E+00      0.852614  0.000000E+00      0.852614  0.000000E+00  0.000000E+00      -1.70523       85.7100       1.33362        1       Scatag_D
      50       1720.40       5538.98  0.000000E+00  0.000000E+00       2621.18  0.000000E+00       2457.88       6654.73  0.000000E+00       11007.3       39.5491       10888.7 -1.000000E+10 -1.000000E+10 -1.000000E+10 -1.000000E+10 -1.000000E+10  0.000000E+00  0.000000E+00  0.000000E+00  0.000000E+00  0.000000E+00  0.000000E+00  0.000000E+00  0.000000E+00  0.000000E+00  0.000000E+00  0.000000E+00  0.000000E+00       Funtag_D
      51  3.918624E+05  3.509391E+05       40923.2       11.5034     0.0868110      0.986769      0.193445      0.228504     0.0164978   -0.00932596    0.00782854       1.00729       Opttag_D
      51       84.8716       1.44670       1.00000  0.000000E+00       1.00000  0.000000E+00      0.927469       73.5247       1.00000       25.5366  0.000000E+00      0.850160  0.000000E+00      0.850160  0.000000E+00  0.000000E+00      -1.70032       85.7100       1.33362        1       Scatag_D
      51       1720.04       5533.39  0.000000E+00  0.000000E+00       2622.17  0.000000E+00       2457.54       6654.13  0.000000E+00       11010.6       39.5050       10885.8 -1.000000E+10 -1.000000E+10 -1.000000E+10 -1.000000E+10 -1.000000E+10  0.000000E+00  0.000000E+00  0.000000E+00  0.000000E+00  0.000000E+00  0.000000E+00  0.000000E+00  0.000000E+00  0.000000E+00  0.000000E+00  0.000000E+00  0.000000E+00       Funtag_D
      52  3.918126E+05  3.508725E+05       40940.1       12.2015     0.0882101      0.994136      0.193384      0.228465     0.0166630   -0.00944258    0.00783342       1.00723       Opttag_D
      52       84.9247       1.44846       1.00000  0.000000E+00       1.00000  0.000000E+00      0.927492       73.5803       1.00000       25.5347  0.000000E+00      0.847035  0.000000E+00      0.847035  0.000000E+00  0.000000E+00      -1.69407       85.7100       1.33362        1       Scatag_D
      52       1722.19       5532.72  0.000000E+00  0.000000E+00       2623.43  0.000000E+00       2457.74       6666.46  0.000000E+00       11015.7       39.4696       10882.3 -1.000000E+10 -1.000000E+10 -1.000000E+10 -1.000000E+10 -1.000000E+10  0.000000E+00  0.000000E+00  0.000000E+00  0.000000E+00  0.000000E+00  0.000000E+00  0.000000E+00  0.000000E+00  0.000000E+00  0.000000E+00  0.000000E+00  0.000000E+00       Funtag_D
      53  3.917795E+05  3.507600E+05       41019.5       26.8899     0.0904825       1.00507      0.193288      0.228415     0.0169419   -0.00965383    0.00785057       1.00744       Opttag_D
      53       85.0076       1.45073       1.00000  0.000000E+00       1.00000  0.000000E+00      0.927524       73.6568       1.00000       25.5326  0.000000E+00      0.842298  0.000000E+00      0.842298  0.000000E+00  0.000000E+00      -1.68460       85.7100       1.33362        1       Scatag_D
      53       1729.74       5535.13  0.000000E+00  0.000000E+00       2625.54  0.000000E+00       2459.67       6727.32  0.000000E+00       11025.2       39.3535       10877.6 -1.000000E+10 -1.000000E+10 -1.000000E+10 -1.000000E+10 -1.000000E+10  0.000000E+00  0.000000E+00  0.000000E+00  0.000000E+00  0.000000E+00  0.000000E+00  0.000000E+00  0.000000E+00  0.000000E+00  0.000000E+00  0.000000E+00  0.000000E+00       Funtag_D
      54  3.917182E+05  3.507237E+05       40994.5       12.9463     0.0912143       1.00829      0.193256      0.228394     0.0170318   -0.00971981    0.00784999       1.00678       Opttag_D
      54       85.0335       1.45132       1.00000  0.000000E+00       1.00000  0.000000E+00      0.927530       73.6799       1.00000       25.5317  0.000000E+00      0.840632  0.000000E+00      0.840632  0.000000E+00  0.000000E+00      -1.68126       85.7100       1.33362        1       Scatag_D
      54       1729.48       5527.81  0.000000E+00  0.000000E+00       2626.12  0.000000E+00       2460.62       6707.01  0.000000E+00       11028.3       39.3138       10875.8 -1.000000E+10 -1.000000E+10 -1.000000E+10 -1.000000E+10 -1.000000E+10  0.000000E+00  0.000000E+00  0.000000E+00  0.000000E+00  0.000000E+00  0.000000E+00  0.000000E+00  0.000000E+00  0.000000E+00  0.000000E+00  0.000000E+00  0.000000E+00       Funtag_D
      55  3.916827E+05  3.506962E+05       40986.4       9.84572     0.0915105       1.00904      0.193237      0.228385     0.0171000   -0.00974325    0.00784988       1.00662       Opttag_D
      55       85.0422       1.45090       1.00000  0.000000E+00       1.00000  0.000000E+00      0.927528       73.6803       1.00000       25.5295  0.000000E+00      0.839794  0.000000E+00      0.839794  0.000000E+00  0.000000E+00      -1.67959       85.7100       1.33362        1       Scatag_D
      55       1729.43       5526.08  0.000000E+00  0.000000E+00       2626.30  0.000000E+00       2461.47       6700.29  0.000000E+00       11029.0       39.2418       10874.7 -1.000000E+10 -1.000000E+10 -1.000000E+10 -1.000000E+10 -1.000000E+10  0.000000E+00  0.000000E+00  0.000000E+00  0.000000E+00  0.000000E+00  0.000000E+00  0.000000E+00  0.000000E+00  0.000000E+00  0.000000E+00  0.000000E+00  0.000000E+00       Funtag_D
      56  3.916292E+05  3.506239E+05       41005.3       13.8034     0.0925491       1.01236      0.193186      0.228354     0.0172791   -0.00984800    0.00785768       1.00681       Opttag_D
      56       85.0742       1.45046       1.00000  0.000000E+00       1.00000  0.000000E+00      0.927525       73.6905       1.00000       25.5249  0.000000E+00      0.837442  0.000000E+00      0.837442  0.000000E+00  0.000000E+00      -1.67488       85.7100       1.33362        1       Scatag_D
      56       1732.87       5528.11  0.000000E+00  0.000000E+00       2627.06  0.000000E+00       2463.91       6709.36  0.000000E+00       11032.5       39.0706       10872.5 -1.000000E+10 -1.000000E+10 -1.000000E+10 -1.000000E+10 -1.000000E+10  0.000000E+00  0.000000E+00  0.000000E+00  0.000000E+00  0.000000E+00  0.000000E+00  0.000000E+00  0.000000E+00  0.000000E+00  0.000000E+00  0.000000E+00  0.000000E+00       Funtag_D
      57  3.915767E+05  3.505164E+05       41060.3       22.2198     0.0943705       1.01799      0.193108      0.228310     0.0175458    -0.0100443    0.00787340       1.00706       Opttag_D
      57       85.1278       1.44994       1.00000  0.000000E+00       1.00000  0.000000E+00      0.927511       73.7011       1.00000       25.5190  0.000000E+00      0.833752  0.000000E+00      0.833752  0.000000E+00  0.000000E+00      -1.66750       85.7100       1.33362        1       Scatag_D
      57       1739.81       5530.86  0.000000E+00  0.000000E+00       2628.48  0.000000E+00       2467.74       6744.21  0.000000E+00       11039.6       38.8353       10870.8 -1.000000E+10 -1.000000E+10 -1.000000E+10 -1.000000E+10 -1.000000E+10  0.000000E+00  0.000000E+00  0.000000E+00  0.000000E+00  0.000000E+00  0.000000E+00  0.000000E+00  0.000000E+00  0.000000E+00  0.000000E+00  0.000000E+00  0.000000E+00       Funtag_D
      58  3.915161E+05  3.504547E+05       41061.3       12.6300     0.0956605       1.02209      0.193062      0.228279     0.0176985    -0.0102116    0.00788176       1.00698       Opttag_D
      58       85.1654       1.44997       1.00000  0.000000E+00       1.00000  0.000000E+00      0.927498       73.7087       1.00000       25.5179  0.000000E+00      0.831435  0.000000E+00      0.831435  0.000000E+00  0.000000E+00      -1.66287       85.7100       1.33362        1       Scatag_D
      58       1743.51       5530.07  0.000000E+00  0.000000E+00       2629.40  0.000000E+00       2470.25       6734.32  0.000000E+00       11044.9       38.7495       10870.1 -1.000000E+10 -1.000000E+10 -1.000000E+10 -1.000000E+10 -1.000000E+10  0.000000E+00  0.000000E+00  0.000000E+00  0.000000E+00  0.000000E+00  0.000000E+00  0.000000E+00  0.000000E+00  0.000000E+00  0.000000E+00  0.000000E+00  0.000000E+00       Funtag_D
      59  3.914744E+05  3.504201E+05       41054.3       8.76109     0.0965176       1.02465      0.193036      0.228255     0.0177844    -0.0103157    0.00788415       1.00707       Opttag_D
      59       85.1886       1.44993       1.00000  0.000000E+00       1.00000  0.000000E+00      0.927481       73.7063       1.00000       25.5189  0.000000E+00      0.829889  0.000000E+00      0.829889  0.000000E+00  0.000000E+00      -1.65978       85.7100       1.33362        1       Scatag_D
      59       1744.56       5531.06  0.000000E+00  0.000000E+00       2629.93  0.000000E+00       2470.45       6722.70  0.000000E+00       11048.2       38.7518       10868.6 -1.000000E+10 -1.000000E+10 -1.000000E+10 -1.000000E+10 -1.000000E+10  0.000000E+00  0.000000E+00  0.000000E+00  0.000000E+00  0.000000E+00  0.000000E+00  0.000000E+00  0.000000E+00  0.000000E+00  0.000000E+00  0.000000E+00  0.000000E+00       Funtag_D
      60  3.914367E+05  3.503847E+05       41052.0       12.1238     0.0975882       1.02763      0.193015      0.228229     0.0178722    -0.0104608    0.00788770       1.00738       Opttag_D
      60       85.2162       1.44984       1.00000  0.000000E+00       1.00000  0.000000E+00      0.927454       73.6961       1.00000       25.5226  0.000000E+00      0.827499  0.000000E+00      0.827499  0.000000E+00  0.000000E+00      -1.65500       85.7100       1.33362        1       Scatag_D
      60       1746.13       5534.41  0.000000E+00  0.000000E+00       2630.49  0.000000E+00       2469.79       6714.51  0.000000E+00       11051.9       38.8080       10866.0 -1.000000E+10 -1.000000E+10 -1.000000E+10 -1.000000E+10 -1.000000E+10  0.000000E+00  0.000000E+00  0.000000E+00  0.000000E+00  0.000000E+00  0.000000E+00  0.000000E+00  0.000000E+00  0.000000E+00  0.000000E+00  0.000000E+00  0.000000E+00       Funtag_D
      61  3.913984E+05  3.503479E+05       41050.5       14.3830     0.0990136       1.03144      0.192993      0.228201     0.0179633    -0.0106533    0.00789106       1.00781       Opttag_D
      61       85.2521       1.44987       1.00000  0.000000E+00       1.00000  0.000000E+00      0.927417       73.6769       1.00000       25.5309  0.000000E+00      0.823834  0.000000E+00      0.823834  0.000000E+00  0.000000E+00      -1.64767       85.7100       1.33362        1       Scatag_D
      61       1747.62       5539.19  0.000000E+00  0.000000E+00       2631.19  0.000000E+00       2469.16       6705.28  0.000000E+00       11056.9       38.8831       10862.2 -1.000000E+10 -1.000000E+10 -1.000000E+10 -1.000000E+10 -1.000000E+10  0.000000E+00  0.000000E+00  0.000000E+00  0.000000E+00  0.000000E+00  0.000000E+00  0.000000E+00  0.000000E+00  0.000000E+00  0.000000E+00  0.000000E+00  0.000000E+00       Funtag_D
      62  3.913604E+05  3.503390E+05       41021.4       10.5372     0.0997016       1.03306      0.192990      0.228194     0.0179855    -0.0107581    0.00789009       1.00772       Opttag_D
      62       85.2681       1.44986       1.00000  0.000000E+00       1.00000  0.000000E+00      0.927395       73.6581       1.00000       25.5374  0.000000E+00      0.821522  0.000000E+00      0.821522  0.000000E+00  0.000000E+00      -1.64304       85.7100       1.33362        1       Scatag_D
      62       1747.19       5538.12  0.000000E+00  0.000000E+00       2631.42  0.000000E+00       2468.56       6679.38  0.000000E+00       11058.6       38.8845       10859.2 -1.000000E+10 -1.000000E+10 -1.000000E+10 -1.000000E+10 -1.000000E+10  0.000000E+00  0.000000E+00  0.000000E+00  0.000000E+00  0.000000E+00  0.000000E+00  0.000000E+00  0.000000E+00  0.000000E+00  0.000000E+00  0.000000E+00  0.000000E+00       Funtag_D
      63  3.913101E+05  3.503335E+05       40976.7       10.4070      0.100878       1.03570      0.192994      0.228191     0.0179992    -0.0109796    0.00788683       1.00782       Opttag_D
      63       85.2945       1.44996       1.00000  0.000000E+00       1.00000  0.000000E+00      0.927359       73.6188       1.00000       25.5503  0.000000E+00      0.817294  0.000000E+00      0.817294  0.000000E+00  0.000000E+00      -1.63459       85.7100       1.33362        1       Scatag_D
      63       1745.75       5539.22  0.000000E+00  0.000000E+00       2631.84  0.000000E+00       2468.62       6637.86  0.000000E+00       11060.6       38.8011       10853.9 -1.000000E+10 -1.000000E+10 -1.000000E+10 -1.000000E+10 -1.000000E+10  0.000000E+00  0.000000E+00  0.000000E+00  0.000000E+00  0.000000E+00  0.000000E+00  0.000000E+00  0.000000E+00  0.000000E+00  0.000000E+00  0.000000E+00  0.000000E+00       Funtag_D
      64  3.912761E+05  3.503339E+05       40942.2       14.2652      0.102069       1.03844      0.193002      0.228182     0.0179980    -0.0112197    0.00788218       1.00792       Opttag_D
      64       85.3215       1.45034       1.00000  0.000000E+00       1.00000  0.000000E+00      0.927326       73.5815       1.00000       25.5621  0.000000E+00      0.813795  0.000000E+00      0.813795  0.000000E+00  0.000000E+00      -1.62759       85.7100       1.33362        1       Scatag_D
      64       1743.69       5540.32  0.000000E+00  0.000000E+00       2632.34  0.000000E+00       2470.59       6604.44  0.000000E+00       11062.8       38.6418       10849.4 -1.000000E+10 -1.000000E+10 -1.000000E+10 -1.000000E+10 -1.000000E+10  0.000000E+00  0.000000E+00  0.000000E+00  0.000000E+00  0.000000E+00  0.000000E+00  0.000000E+00  0.000000E+00  0.000000E+00  0.000000E+00  0.000000E+00  0.000000E+00       Funtag_D
      65  3.912403E+05  3.503360E+05       40904.3       9.96409      0.102840       1.04021      0.193005      0.228179     0.0179929    -0.0113831    0.00787791       1.00777       Opttag_D
      65       85.3385       1.45058       1.00000  0.000000E+00       1.00000  0.000000E+00      0.927307       73.5539       1.00000       25.5698  0.000000E+00      0.811995  0.000000E+00      0.811995  0.000000E+00  0.000000E+00      -1.62399       85.7100       1.33362        1       Scatag_D
      65       1741.80       5538.72  0.000000E+00  0.000000E+00       2632.77  0.000000E+00       2472.77       6569.59  0.000000E+00       11063.8       38.5041       10846.4 -1.000000E+10 -1.000000E+10 -1.000000E+10 -1.000000E+10 -1.000000E+10  0.000000E+00  0.000000E+00  0.000000E+00  0.000000E+00  0.000000E+00  0.000000E+00  0.000000E+00  0.000000E+00  0.000000E+00  0.000000E+00  0.000000E+00  0.000000E+00       Funtag_D
      66  3.912052E+05  3.503367E+05       40868.6       8.58476      0.103885       1.04273      0.193007      0.228176     0.0179913    -0.0116179    0.00787290       1.00766       Opttag_D
      66       85.3618       1.45091       1.00000  0.000000E+00       1.00000  0.000000E+00      0.927283       73.5196       1.00000       25.5789  0.000000E+00      0.810119  0.000000E+00      0.810119  0.000000E+00  0.000000E+00      -1.62024       85.7100       1.33362        1       Scatag_D
      66       1739.59       5537.46  0.000000E+00  0.000000E+00       2633.45  0.000000E+00       2476.22       6535.79  0.000000E+00       11065.2       38.3676       10842.5 -1.000000E+10 -1.000000E+10 -1.000000E+10 -1.000000E+10 -1.000000E+10  0.000000E+00  0.000000E+00  0.000000E+00  0.000000E+00  0.000000E+00  0.000000E+00  0.000000E+00  0.000000E+00  0.000000E+00  0.000000E+00  0.000000E+00  0.000000E+00       Funtag_D
      67  3.911805E+05  3.503318E+05       40848.8       10.1086      0.104712       1.04477      0.193004      0.228174     0.0180034    -0.0117766    0.00787471       1.00749       Opttag_D
      67       85.3804       1.45099       1.00000  0.000000E+00       1.00000  0.000000E+00      0.927264       73.4929       1.00000       25.5867  0.000000E+00      0.808985  0.000000E+00      0.808985  0.000000E+00  0.000000E+00      -1.61797       85.7100       1.33362        1       Scatag_D
      67       1740.39       5535.62  0.000000E+00  0.000000E+00       2634.03  0.000000E+00       2478.56       6515.83  0.000000E+00       11066.4       38.3248       10839.6 -1.000000E+10 -1.000000E+10 -1.000000E+10 -1.000000E+10 -1.000000E+10  0.000000E+00  0.000000E+00  0.000000E+00  0.000000E+00  0.000000E+00  0.000000E+00  0.000000E+00  0.000000E+00  0.000000E+00  0.000000E+00  0.000000E+00  0.000000E+00       Funtag_D
      68  3.911482E+05  3.503240E+05       40824.3       9.45718      0.105749       1.04738      0.192999      0.228174     0.0180227    -0.0119697    0.00787851       1.00732       Opttag_D
      68       85.4040       1.45103       1.00000  0.000000E+00       1.00000  0.000000E+00      0.927240       73.4602       1.00000       25.5971  0.000000E+00      0.807600  0.000000E+00      0.807600  0.000000E+00  0.000000E+00      -1.61520       85.7100       1.33362        1       Scatag_D
      68       1742.07       5533.75  0.000000E+00  0.000000E+00       2634.82  0.000000E+00       2480.97       6489.64  0.000000E+00       11068.4       38.3386       10836.2 -1.000000E+10 -1.000000E+10 -1.000000E+10 -1.000000E+10 -1.000000E+10  0.000000E+00  0.000000E+00  0.000000E+00  0.000000E+00  0.000000E+00  0.000000E+00  0.000000E+00  0.000000E+00  0.000000E+00  0.000000E+00  0.000000E+00  0.000000E+00       Funtag_D
      69  3.911176E+05  3.503095E+05       40808.1       16.7226      0.107944       1.05371      0.192986      0.228201     0.0180586    -0.0124024    0.00789121       1.00759       Opttag_D
      69       85.4544       1.45109       1.00000  0.000000E+00       1.00000  0.000000E+00      0.927190       73.3933       1.00000       25.6227  0.000000E+00      0.804417  0.000000E+00      0.804417  0.000000E+00  0.000000E+00      -1.60883       85.7100       1.33362        1       Scatag_D
      69       1747.69       5536.75  0.000000E+00  0.000000E+00       2636.48  0.000000E+00       2484.94       6458.98  0.000000E+00       11074.2       38.4890       10830.5 -1.000000E+10 -1.000000E+10 -1.000000E+10 -1.000000E+10 -1.000000E+10  0.000000E+00  0.000000E+00  0.000000E+00  0.000000E+00  0.000000E+00  0.000000E+00  0.000000E+00  0.000000E+00  0.000000E+00  0.000000E+00  0.000000E+00  0.000000E+00       Funtag_D
      70  3.910840E+05  3.503035E+05       40780.5       8.31184      0.108426       1.05896      0.192982      0.228204     0.0180735    -0.0124777    0.00789112       1.00726       Opttag_D
      70       85.4660       1.45102       1.00000  0.000000E+00       1.00000  0.000000E+00      0.927179       73.3787       1.00000       25.6287  0.000000E+00      0.803541  0.000000E+00      0.803541  0.000000E+00  0.000000E+00      -1.60708       85.7100       1.33362        1       Scatag_D
      70       1747.65       5533.11  0.000000E+00  0.000000E+00       2636.87  0.000000E+00       2484.85       6435.20  0.000000E+00       11075.5       38.4963       10828.9 -1.000000E+10 -1.000000E+10 -1.000000E+10 -1.000000E+10 -1.000000E+10  0.000000E+00  0.000000E+00  0.000000E+00  0.000000E+00  0.000000E+00  0.000000E+00  0.000000E+00  0.000000E+00  0.000000E+00  0.000000E+00  0.000000E+00  0.000000E+00       Funtag_D
      71  3.910691E+05  3.502963E+05       40772.8       7.17387      0.108460       1.05914      0.192979      0.228203     0.0180913    -0.0124878    0.00788908       1.00721       Opttag_D
      71       85.4674       1.45080       1.00000  0.000000E+00       1.00000  0.000000E+00      0.927178       73.3770       1.00000       25.6291  0.000000E+00      0.803189  0.000000E+00      0.803189  0.000000E+00  0.000000E+00      -1.60638       85.7100       1.33362        1       Scatag_D
      71       1746.75       5532.58  0.000000E+00  0.000000E+00       2636.89  0.000000E+00       2483.67       6431.19  0.000000E+00       11075.3       38.4942       10827.9 -1.000000E+10 -1.000000E+10 -1.000000E+10 -1.000000E+10 -1.000000E+10  0.000000E+00  0.000000E+00  0.000000E+00  0.000000E+00  0.000000E+00  0.000000E+00  0.000000E+00  0.000000E+00  0.000000E+00  0.000000E+00  0.000000E+00  0.000000E+00       Funtag_D
      72  3.910449E+05  3.502819E+05       40763.0       8.14548      0.109037       1.06490      0.192972      0.228210     0.0181270    -0.0126266    0.00788705       1.00721       Opttag_D
      72       85.4828       1.45058       1.00000  0.000000E+00       1.00000  0.000000E+00      0.927169       73.3601       1.00000       25.6352  0.000000E+00      0.801555  0.000000E+00      0.801555  0.000000E+00  0.000000E+00      -1.60311       85.7100       1.33362        1       Scatag_D
      72       1745.85       5532.52  0.000000E+00  0.000000E+00       2637.31  0.000000E+00       2482.19       6424.84  0.000000E+00       11076.7       38.5086       10825.1 -1.000000E+10 -1.000000E+10 -1.000000E+10 -1.000000E+10 -1.000000E+10  0.000000E+00  0.000000E+00  0.000000E+00  0.000000E+00  0.000000E+00  0.000000E+00  0.000000E+00  0.000000E+00  0.000000E+00  0.000000E+00  0.000000E+00  0.000000E+00       Funtag_D
      73  3.910347E+05  3.502662E+05       40768.5       17.8380      0.110678       1.08138      0.192958      0.228243     0.0181660    -0.0130483    0.00789102       1.00757       Opttag_D
      73       85.5248       1.45083       1.00000  0.000000E+00       1.00000  0.000000E+00      0.927142       73.3159       1.00000       25.6541  0.000000E+00      0.797849  0.000000E+00      0.797849  0.000000E+00  0.000000E+00      -1.59570       85.7100       1.33362        1       Scatag_D
      73       1747.61       5536.51  0.000000E+00  0.000000E+00       2638.62  0.000000E+00       2481.87       6422.91  0.000000E+00       11082.1       38.5353       10820.4 -1.000000E+10 -1.000000E+10 -1.000000E+10 -1.000000E+10 -1.000000E+10  0.000000E+00  0.000000E+00  0.000000E+00  0.000000E+00  0.000000E+00  0.000000E+00  0.000000E+00  0.000000E+00  0.000000E+00  0.000000E+00  0.000000E+00  0.000000E+00       Funtag_D
      74  3.910064E+05  3.502663E+05       40740.1       7.14741      0.110794       1.08248      0.192959      0.228242     0.0181656    -0.0130733    0.00788657       1.00723       Opttag_D
      74       85.5279       1.45093       1.00000  0.000000E+00       1.00000  0.000000E+00      0.927142       73.3129       1.00000       25.6550  0.000000E+00      0.797568  0.000000E+00      0.797568  0.000000E+00  0.000000E+00      -1.59514       85.7100       1.33362        1       Scatag_D
      74       1745.64       5532.77  0.000000E+00  0.000000E+00       2638.70  0.000000E+00       2482.02       6400.17  0.000000E+00       11082.5       38.5342       10819.8 -1.000000E+10 -1.000000E+10 -1.000000E+10 -1.000000E+10 -1.000000E+10  0.000000E+00  0.000000E+00  0.000000E+00  0.000000E+00  0.000000E+00  0.000000E+00  0.000000E+00  0.000000E+00  0.000000E+00  0.000000E+00  0.000000E+00  0.000000E+00       Funtag_D
      75  3.909920E+05  3.502658E+05       40726.2       5.51300      0.111336       1.08779      0.192958      0.228253     0.0181670    -0.0132053    0.00788488       1.00717       Opttag_D
      75       85.5419       1.45124       1.00000  0.000000E+00       1.00000  0.000000E+00      0.927136       73.2994       1.00000       25.6611  0.000000E+00      0.796430  0.000000E+00      0.796430  0.000000E+00  0.000000E+00      -1.59286       85.7100       1.33362        1       Scatag_D
      75       1744.89       5532.13  0.000000E+00  0.000000E+00       2639.13  0.000000E+00       2482.49       6386.52  0.000000E+00       11084.3       38.5377       10818.2 -1.000000E+10 -1.000000E+10 -1.000000E+10 -1.000000E+10 -1.000000E+10  0.000000E+00  0.000000E+00  0.000000E+00  0.000000E+00  0.000000E+00  0.000000E+00  0.000000E+00  0.000000E+00  0.000000E+00  0.000000E+00  0.000000E+00  0.000000E+00       Funtag_D
      76  3.909762E+05  3.502625E+05       40713.7       6.57851      0.112298       1.09688      0.192955      0.228273     0.0181752    -0.0134410    0.00788468       1.00713       Opttag_D
      76       85.5672       1.45171       1.00000  0.000000E+00       1.00000  0.000000E+00      0.927123       73.2761       1.00000       25.6729  0.000000E+00      0.794293  0.000000E+00      0.794293  0.000000E+00  0.000000E+00      -1.58859       85.7100       1.33362        1       Scatag_D
      76       1744.80       5531.67  0.000000E+00  0.000000E+00       2639.93  0.000000E+00       2482.78       6372.64  0.000000E+00       11087.7       38.5606       10815.7 -1.000000E+10 -1.000000E+10 -1.000000E+10 -1.000000E+10 -1.000000E+10  0.000000E+00  0.000000E+00  0.000000E+00  0.000000E+00  0.000000E+00  0.000000E+00  0.000000E+00  0.000000E+00  0.000000E+00  0.000000E+00  0.000000E+00  0.000000E+00       Funtag_D
      77  3.909557E+05  3.502510E+05       40704.7       12.0317      0.113870       1.11074      0.192947      0.228310     0.0182035    -0.0138257    0.00788705       1.00721       Opttag_D
      77       85.6106       1.45243       1.00000  0.000000E+00       1.00000  0.000000E+00      0.927104       73.2412       1.00000       25.6941  0.000000E+00      0.790605  0.000000E+00      0.790605  0.000000E+00  0.000000E+00      -1.58121       85.7100       1.33362        1       Scatag_D
      77       1745.85       5532.53  0.000000E+00  0.000000E+00       2641.27  0.000000E+00       2482.39       6359.59  0.000000E+00       11093.0       38.6260       10811.4 -1.000000E+10 -1.000000E+10 -1.000000E+10 -1.000000E+10 -1.000000E+10  0.000000E+00  0.000000E+00  0.000000E+00  0.000000E+00  0.000000E+00  0.000000E+00  0.000000E+00  0.000000E+00  0.000000E+00  0.000000E+00  0.000000E+00  0.000000E+00       Funtag_D
      78  3.909318E+05  3.502428E+05       40689.0       6.09561      0.115245       1.12225      0.192938      0.228344     0.0182239    -0.0141688    0.00788844       1.00699       Opttag_D
      78       85.6493       1.45316       1.00000  0.000000E+00       1.00000  0.000000E+00      0.927087       73.2133       1.00000       25.7138  0.000000E+00      0.787390  0.000000E+00      0.787390  0.000000E+00  0.000000E+00      -1.57478       85.7100       1.33362        1       Scatag_D
      78       1746.46       5530.09  0.000000E+00  0.000000E+00       2642.55  0.000000E+00       2481.94       6343.19  0.000000E+00       11098.0       38.6716       10808.1 -1.000000E+10 -1.000000E+10 -1.000000E+10 -1.000000E+10 -1.000000E+10  0.000000E+00  0.000000E+00  0.000000E+00  0.000000E+00  0.000000E+00  0.000000E+00  0.000000E+00  0.000000E+00  0.000000E+00  0.000000E+00  0.000000E+00  0.000000E+00       Funtag_D
      79  3.909153E+05  3.502297E+05       40685.6       5.64436      0.115550       1.12396      0.192930      0.228344     0.0182564    -0.0142381    0.00788783       1.00689       Opttag_D
      79       85.6604       1.45314       1.00000  0.000000E+00       1.00000  0.000000E+00      0.927084       73.2095       1.00000       25.7188  0.000000E+00      0.786261  0.000000E+00      0.786261  0.000000E+00  0.000000E+00      -1.57252       85.7100       1.33362        1       Scatag_D
      79       1746.19       5529.04  0.000000E+00  0.000000E+00       2642.83  0.000000E+00       2481.08       6342.72  0.000000E+00       11098.3       38.6707       10806.8 -1.000000E+10 -1.000000E+10 -1.000000E+10 -1.000000E+10 -1.000000E+10  0.000000E+00  0.000000E+00  0.000000E+00  0.000000E+00  0.000000E+00  0.000000E+00  0.000000E+00  0.000000E+00  0.000000E+00  0.000000E+00  0.000000E+00  0.000000E+00       Funtag_D
      80  3.908916E+05  3.501982E+05       40693.4       9.27302      0.116127       1.12638      0.192911      0.228344     0.0183345    -0.0143839    0.00788623       1.00680       Opttag_D
      80       85.6840       1.45307       1.00000  0.000000E+00       1.00000  0.000000E+00      0.927081       73.2053       1.00000       25.7287  0.000000E+00      0.783667  0.000000E+00      0.783667  0.000000E+00  0.000000E+00      -1.56733       85.7100       1.33362        1       Scatag_D
      80       1745.48       5528.09  0.000000E+00  0.000000E+00       2643.34  0.000000E+00       2479.48       6355.44  0.000000E+00       11098.7       38.6638       10804.3 -1.000000E+10 -1.000000E+10 -1.000000E+10 -1.000000E+10 -1.000000E+10  0.000000E+00  0.000000E+00  0.000000E+00  0.000000E+00  0.000000E+00  0.000000E+00  0.000000E+00  0.000000E+00  0.000000E+00  0.000000E+00  0.000000E+00  0.000000E+00       Funtag_D
      81  3.908776E+05  3.501669E+05       40710.7       12.4191      0.116848       1.12948      0.192891      0.228334     0.0184121    -0.0145480    0.00788536       1.00679       Opttag_D
      81       85.7126       1.45319       1.00000  0.000000E+00       1.00000  0.000000E+00      0.927081       73.1988       1.00000       25.7412  0.000000E+00      0.780512  0.000000E+00      0.780512  0.000000E+00  0.000000E+00      -1.56102       85.7100       1.33362        1       Scatag_D
      81       1745.10       5527.99  0.000000E+00  0.000000E+00       2644.05  0.000000E+00       2478.78       6374.30  0.000000E+00       11100.0       38.6635       10801.9 -1.000000E+10 -1.000000E+10 -1.000000E+10 -1.000000E+10 -1.000000E+10  0.000000E+00  0.000000E+00  0.000000E+00  0.000000E+00  0.000000E+00  0.000000E+00  0.000000E+00  0.000000E+00  0.000000E+00  0.000000E+00  0.000000E+00  0.000000E+00       Funtag_D
      82  3.908599E+05  3.501609E+05       40698.9       7.13269      0.116792       1.12842      0.192887      0.228329     0.0184269    -0.0145431    0.00788261       1.00670       Opttag_D
      82       85.7130       1.45312       1.00000  0.000000E+00       1.00000  0.000000E+00      0.927085       73.2016       1.00000       25.7404  0.000000E+00      0.780158  0.000000E+00      0.780158  0.000000E+00  0.000000E+00      -1.56032       85.7100       1.33362        1       Scatag_D
      82       1743.88       5526.98  0.000000E+00  0.000000E+00       2643.98  0.000000E+00       2478.92       6365.86  0.000000E+00       11099.3       38.6495       10801.4 -1.000000E+10 -1.000000E+10 -1.000000E+10 -1.000000E+10 -1.000000E+10  0.000000E+00  0.000000E+00  0.000000E+00  0.000000E+00  0.000000E+00  0.000000E+00  0.000000E+00  0.000000E+00  0.000000E+00  0.000000E+00  0.000000E+00  0.000000E+00       Funtag_D
      83  3.908381E+05  3.501419E+05       40696.2       6.00009      0.117105       1.12885      0.192875      0.228318     0.0184741    -0.0146310    0.00787883       1.00666       Opttag_D
      83       85.7281       1.45325       1.00000  0.000000E+00       1.00000  0.000000E+00      0.927095       73.2009       1.00000       25.7459  0.000000E+00      0.778083  0.000000E+00      0.778083  0.000000E+00  0.000000E+00      -1.55617       85.7100       1.33362        1       Scatag_D
      83       1742.21       5526.46  0.000000E+00  0.000000E+00       2644.29  0.000000E+00       2479.42       6366.44  0.000000E+00       11099.3       38.6391       10799.5 -1.000000E+10 -1.000000E+10 -1.000000E+10 -1.000000E+10 -1.000000E+10  0.000000E+00  0.000000E+00  0.000000E+00  0.000000E+00  0.000000E+00  0.000000E+00  0.000000E+00  0.000000E+00  0.000000E+00  0.000000E+00  0.000000E+00  0.000000E+00       Funtag_D
      84  3.908238E+05  3.501221E+05       40701.7       6.77811      0.117507       1.12971      0.192863      0.228308     0.0185230    -0.0147217    0.00787795       1.00664       Opttag_D
      84       85.7460       1.45344       1.00000  0.000000E+00       1.00000  0.000000E+00      0.927104       73.1995       1.00000       25.7534  0.000000E+00      0.775910  0.000000E+00      0.775910  0.000000E+00  0.000000E+00      -1.55182       85.7100       1.33362        1       Scatag_D
      84       1741.82       5526.28  0.000000E+00  0.000000E+00       2644.74  0.000000E+00       2479.76       6373.21  0.000000E+00       11099.6       38.6471       10797.6 -1.000000E+10 -1.000000E+10 -1.000000E+10 -1.000000E+10 -1.000000E+10  0.000000E+00  0.000000E+00  0.000000E+00  0.000000E+00  0.000000E+00  0.000000E+00  0.000000E+00  0.000000E+00  0.000000E+00  0.000000E+00  0.000000E+00  0.000000E+00       Funtag_D
      85  3.908135E+05  3.500676E+05       40745.9       16.1853      0.118680       1.13153      0.192826      0.228292     0.0186582    -0.0149455    0.00788231       1.00697       Opttag_D
      85       85.7988       1.45410       1.00000  0.000000E+00       1.00000  0.000000E+00      0.927134       73.1984       1.00000       25.7774  0.000000E+00      0.770339  0.000000E+00      0.770339  0.000000E+00  0.000000E+00      -1.54068       85.7100       1.33362        1       Scatag_D
      85       1743.75       5529.90  0.000000E+00  0.000000E+00       2646.12  0.000000E+00       2480.93       6412.15  0.000000E+00       11101.5       38.6937       10792.9 -1.000000E+10 -1.000000E+10 -1.000000E+10 -1.000000E+10 -1.000000E+10  0.000000E+00  0.000000E+00  0.000000E+00  0.000000E+00  0.000000E+00  0.000000E+00  0.000000E+00  0.000000E+00  0.000000E+00  0.000000E+00  0.000000E+00  0.000000E+00       Funtag_D
      86  3.907877E+05  3.500603E+05       40727.4       5.99913      0.118831       1.13152      0.192821      0.228287     0.0186763    -0.0149650    0.00788235       1.00683       Opttag_D
      86       85.8058       1.45420       1.00000  0.000000E+00       1.00000  0.000000E+00      0.927138       73.2000       1.00000       25.7803  0.000000E+00      0.769732  0.000000E+00      0.769732  0.000000E+00  0.000000E+00      -1.53946       85.7100       1.33362        1       Scatag_D
      86       1743.77       5528.42  0.000000E+00  0.000000E+00       2646.38  0.000000E+00       2480.91       6395.65  0.000000E+00       11101.4       38.6879       10792.1 -1.000000E+10 -1.000000E+10 -1.000000E+10 -1.000000E+10 -1.000000E+10  0.000000E+00  0.000000E+00  0.000000E+00  0.000000E+00  0.000000E+00  0.000000E+00  0.000000E+00  0.000000E+00  0.000000E+00  0.000000E+00  0.000000E+00  0.000000E+00       Funtag_D
      87  3.907786E+05  3.500551E+05       40723.5       4.86425      0.118846       1.13102      0.192817      0.228283     0.0186892    -0.0149474    0.00788371       1.00684       Opttag_D
      87       85.8075       1.45413       1.00000  0.000000E+00       1.00000  0.000000E+00      0.927139       73.2021       1.00000       25.7808  0.000000E+00      0.769835  0.000000E+00      0.769835  0.000000E+00  0.000000E+00      -1.53967       85.7100       1.33362        1       Scatag_D
      87       1744.37       5528.45  0.000000E+00  0.000000E+00       2646.48  0.000000E+00       2480.67       6392.31  0.000000E+00       11100.9       38.6741       10791.7 -1.000000E+10 -1.000000E+10 -1.000000E+10 -1.000000E+10 -1.000000E+10  0.000000E+00  0.000000E+00  0.000000E+00  0.000000E+00  0.000000E+00  0.000000E+00  0.000000E+00  0.000000E+00  0.000000E+00  0.000000E+00  0.000000E+00  0.000000E+00       Funtag_D
      88  3.907642E+05  3.500342E+05       40730.0       5.72681      0.119143       1.13012      0.192802      0.228272     0.0187411    -0.0149711    0.00788859       1.00694       Opttag_D
      88       85.8230       1.45420       1.00000  0.000000E+00       1.00000  0.000000E+00      0.927148       73.2073       1.00000       25.7875  0.000000E+00      0.768920  0.000000E+00      0.768920  0.000000E+00  0.000000E+00      -1.53784       85.7100       1.33362        1       Scatag_D
      88       1746.53       5529.59  0.000000E+00  0.000000E+00       2647.07  0.000000E+00       2480.51       6396.68  0.000000E+00       11100.6       38.6429       10790.3 -1.000000E+10 -1.000000E+10 -1.000000E+10 -1.000000E+10 -1.000000E+10  0.000000E+00  0.000000E+00  0.000000E+00  0.000000E+00  0.000000E+00  0.000000E+00  0.000000E+00  0.000000E+00  0.000000E+00  0.000000E+00  0.000000E+00  0.000000E+00       Funtag_D
      89  3.907596E+05  3.500005E+05       40759.1       14.9919      0.119726       1.12833      0.192779      0.228267     0.0188247    -0.0150813    0.00789689       1.00720       Opttag_D
      89       85.8526       1.45440       1.00000  0.000000E+00       1.00000  0.000000E+00      0.927166       73.2128       1.00000       25.8010  0.000000E+00      0.766968  0.000000E+00      0.766968  0.000000E+00  0.000000E+00      -1.53394       85.7100       1.33362        1       Scatag_D
      89       1750.21       5532.39  0.000000E+00  0.000000E+00       2648.35  0.000000E+00       2480.18       6419.69  0.000000E+00       11101.1       38.5727       10788.6 -1.000000E+10 -1.000000E+10 -1.000000E+10 -1.000000E+10 -1.000000E+10  0.000000E+00  0.000000E+00  0.000000E+00  0.000000E+00  0.000000E+00  0.000000E+00  0.000000E+00  0.000000E+00  0.000000E+00  0.000000E+00  0.000000E+00  0.000000E+00       Funtag_D
      90  3.907413E+05  3.499940E+05       40747.4       5.88932      0.119884       1.12811      0.192775      0.228262     0.0188407    -0.0151055    0.00789721       1.00711       Opttag_D
      90       85.8601       1.45451       1.00000  0.000000E+00       1.00000  0.000000E+00      0.927171       73.2133       1.00000       25.8048  0.000000E+00      0.766381  0.000000E+00      0.766381  0.000000E+00  0.000000E+00      -1.53276       85.7100       1.33362        1       Scatag_D
      90       1750.35       5531.42  0.000000E+00  0.000000E+00       2648.63  0.000000E+00       2480.18       6408.33  0.000000E+00       11101.7       38.5669       10788.2 -1.000000E+10 -1.000000E+10 -1.000000E+10 -1.000000E+10 -1.000000E+10  0.000000E+00  0.000000E+00  0.000000E+00  0.000000E+00  0.000000E+00  0.000000E+00  0.000000E+00  0.000000E+00  0.000000E+00  0.000000E+00  0.000000E+00  0.000000E+00       Funtag_D
      91  3.907336E+05  3.499863E+05       40747.3       3.95532      0.120079       1.13017      0.192770      0.228263     0.0188598    -0.0151567    0.00789747       1.00708       Opttag_D
      91       85.8693       1.45461       1.00000  0.000000E+00       1.00000  0.000000E+00      0.927177       73.2121       1.00000       25.8097  0.000000E+00      0.765576  0.000000E+00      0.765576  0.000000E+00  0.000000E+00      -1.53115       85.7100       1.33362        1       Scatag_D
      91       1750.47       5531.16  0.000000E+00  0.000000E+00       2649.04  0.000000E+00       2479.95       6408.03  0.000000E+00       11102.3       38.5547       10787.8 -1.000000E+10 -1.000000E+10 -1.000000E+10 -1.000000E+10 -1.000000E+10  0.000000E+00  0.000000E+00  0.000000E+00  0.000000E+00  0.000000E+00  0.000000E+00  0.000000E+00  0.000000E+00  0.000000E+00  0.000000E+00  0.000000E+00  0.000000E+00       Funtag_D

   MaxLik Scaling Cycle 0097

   Wilson plot

   Statistics in resolution bins after refinement cycle 0003
```


|  |  |
| --- | --- |
|  | - Rxpct factors plot- Rxpct factors table- <Log-Lik Gain>- Ln Sigma A- Correlation Coefficients |


```
   Iters     Tot_Funct    Xray_Funct    Geom_Funct          Grms     rmsD2init     maxD2init             R         Rfree           LLG       LLGfree     rms(bond)    rms(angle)
   Iters             K   B_IMPF_FRAG        K_MISS   B_TEMP_MISS   K_IMPF_MISS   B_IMPF_MISS        K_SOLV   B_TEMP_SOLV   K_IMPF_SOLV   B_IMPF_SOLV        B_TEMP     B_TEMP_11     B_TEMP_12     B_TEMP_22     B_TEMP_13     B_TEMP_23     B_TEMP_33      K_WILSON      B_WILSON      FGCYCLES
   Iters          BOND         ANGLE      IMPROPER       TORSION    SINTORSION      TRIGONAL        PLANAR  BCORRELATION       CONTACT     IDEALDIST        CHIRAL    SIMILARITY       OCCSSUM  UTILDISTANCE     UTILANGLE   UTILTORSION        RHOFIT        ADP-01        ADP-02        ADP-03        ADP-04        ADP-05        ADP-06        ADP-07        ADP-08        ADP-09        ADP-10      RHOFITPC            QM
      92  3.907245E+05  3.499721E+05       40752.4       4.89063      0.120433       1.13448      0.192763      0.228265     0.0188951    -0.0152546    0.00789920       1.00708       Opttag_D
      92       85.8861       1.45475       1.00000  0.000000E+00       1.00000  0.000000E+00      0.927189       73.2090       1.00000       25.8190  0.000000E+00      0.764155  0.000000E+00      0.764155  0.000000E+00  0.000000E+00      -1.52831       85.7100       1.33362        1       Scatag_D
      92       1751.23       5531.11  0.000000E+00  0.000000E+00       2649.84  0.000000E+00       2479.26       6411.86  0.000000E+00       11103.4       38.5419       10787.2 -1.000000E+10 -1.000000E+10 -1.000000E+10 -1.000000E+10 -1.000000E+10  0.000000E+00  0.000000E+00  0.000000E+00  0.000000E+00  0.000000E+00  0.000000E+00  0.000000E+00  0.000000E+00  0.000000E+00  0.000000E+00  0.000000E+00  0.000000E+00       Funtag_D
   Geometry Restraint Deviations SCREEN Information

 

 The refinement has converged on gradient RMS limit (-glim).

 No -bcorrel_zeroocchydrogens_factor flag found - keeping default: will set B for OCC=0 hydrogens to    1.200 times heavy-atom B


 TLS information. Total number of groups:     2


 Group   Natoms    FixRB   FixTLS   Group Tag    Group Spec
     1     2771      Y        Y     tls1    { A|* }
     2     2769      Y        Y     tls2    { B|* }
 
NOTE BUSTER_TLS_SET tls1 { A|* }
NOTE BUSTER_TLS_T   tls1     -2.75683E-01 -1.68119E-01  3.03964E-01 -3.26338E-02  4.03951E-02  3.90114E-02
NOTE BUSTER_TLS_L   tls1      1.42844E+00  1.25261E+00  4.39292E-01  2.96794E-01 -5.41106E-01  2.91932E-01
NOTE BUSTER_TLS_S   tls1      2.84530E-01 -2.02959E-01  2.55729E-01 -2.98009E-02 -1.09482E-01 -1.16911E-01 -1.71883E-02 -9.82972E-02
NOTE BUSTER_TLS_O   tls1     -2.33284E+01 -8.36882E+01  1.12128E+01
NOTE BUSTER_TLS_FIX tls1 ALL
 
NOTE BUSTER_TLS_SET tls2 { B|* }
NOTE BUSTER_TLS_T   tls2     -3.03964E-01 -2.71348E-01  3.03964E-01  6.70261E-03  2.11698E-02 -3.20981E-02
NOTE BUSTER_TLS_L   tls2      1.79743E+00  1.32223E+00  4.53428E-01  4.28487E-01 -6.90721E-01 -7.62929E-01
NOTE BUSTER_TLS_S   tls2      6.31122E-03  1.00071E-01 -1.27050E-01  8.65256E-02  1.54687E-01  3.70038E-02 -7.61979E-02  9.77350E-02
NOTE BUSTER_TLS_O   tls2      2.20360E+01 -7.19286E+01  3.53859E+00
NOTE BUSTER_TLS_FIX tls2 ALL

 For final position, Geometry function summary
     term                           number     weight       rms        GooF      function     fn/numb
     ================================================================================================
     BOND bond lengths (angs)       5655       2.000      0.0079       0.393      1751.23       0.310
     ANGL bond angles (degs)        7677       2.000        1.01       0.600      5531.11       0.720
     TORS split-harmonic tors       1267       0.000       18.91       1.191         0.00       0.000
     SINTOR sinusoidal tors         1267       2.000       18.91                  2649.84       2.091
     PLAN general plane (angs)       962       5.000      0.0144       0.718      2479.26       2.577
     BCORrelations (angs^2)         5644      10.000       0.945       0.337      6411.86       1.136
     CONT (bad contacts)               0       5.000                                 0.00
     IDEAL(ideal-dist contact)      5041       4.000                             11103.40
     CHIRAL (gelly semiharmon)       743       5.000                                38.54
     SIM similarity restraint for NCS and/or TARGET                              10787.20
     OCCSUM restraint on sum of occupanies                                           0.00       as none defined
     DISTAN utility restraints interatomic distance                                  0.00       as none defined
     UTILANGLE utility angle restraints                                              0.00       as none defined
     UTILTOR utility restraints on torsion angles                                    0.00       as none defined
     Peptide omega torsion angles                           2.89 degs - monitor only

 Weighted rms      0.0079         1.007  (temporary rms bond and angle for BUSTER to pick up!)
 Weighted intra-residual rms(bond) for StdProtein (excluding H)      0.0077
 Weighted intra-residual rms(bond) for Buffer     (excluding H)      0.0028
 Outputing geometry progress graph in plotmtv format to file:Geom_Res.mtv

 ############### Running final geometry/screen analysis (screen_final.txt)

 s/r gelly normal termination
```

Statistics and scale factors vs. cycle number
```

|  |  |  |
| --- | --- | --- |
|  | - Rxpct factors- <Log-Lik Gain>- GEOM Residuals- Ln Sigma_A_ | - Scale Factors- B Factors- Imperfection Factors |

```
   Final Geometry Information


---


### 2. Final results

Statistics and scale factors vs. cycle number
```

|  |  |  |
| --- | --- | --- |
|  | - Rxpct factors- <Log-Lik Gain>- GEOM Residuals- Ln Sigma_A_ | - Scale Factors- B Factors- Imperfection Factors |

```
   Average Figure of Merit
   Structure Factor Amplitudes


 # normal termination

  Program stopped.
```

closesok message: time spent in tree = 1203.25 (user) 39.22 (sys)

---

# Normal termination

```
 Resources usage summary:
     Time spent in client ...................  1203.26 (user)       39.24 (system)
     Time spent in scripts (    27 calls) ...     1.86 (user)        0.13 (system)
     Time spent in server ...................     0.00 (user)        0.01 (system)

     Max resident memory used (kb) .......... 433840
     Number of page faults .................. 0 (major)    332704 (minor)
     Number of context switches ............. 2553 (niced)    4664 (other)
```
